# A therapy for suppressing canonical and noncanonical SARS-CoV-2 viral entry and an intrinsic intrapulmonary inflammatory response

**DOI:** 10.1073/pnas.2408109121

**Published:** 2024-07-19

**Authors:** Sandra L. Leibel, Rachael N. McVicar, Rabi Murad, Elizabeth M. Kwong, Alex E. Clark, Asuka Alvarado, Bethany A. Grimmig, Ruslan Nuryyev, Randee E. Young, Jamie C. Lee, Weiqi Peng, Yanfang P. Zhu, Eric Griffis, Cameron J. Nowell, Brian James, Suzie Alarcon, Atul Malhotra, Linden J. Gearing, Paul J. Hertzog, Cheska M. Galapate, Koen M. O. Galenkamp, Cosimo Commisso, Davey M. Smith, Xin Sun, Aaron F. Carlin, Richard L. Sidman, Ben A. Croker, Evan Y. Snyder

**Affiliations:** ^a^Department of Pediatrics, University of California San Diego, La Jolla, CA 92093; ^b^Sanford Consortium for Regenerative Medicine, La Jolla, CA 92037; ^c^Sanford Burnham Prebys Medical Discovery Institute, Center for Stem Cells & Regenerative Medicine, La Jolla, CA 92037; ^d^Department of Medicine, University of California San Diego, La Jolla, CA 92093; ^e^Nikon Imaging Center, University of California San Diego, La Jolla, CA 92093; ^f^Monash Institute of Pharmaceutical Sciences, Parkville, VIC 3052, Australia; ^g^La Jolla Institute for Immunology, La Jolla, CA 92037; ^h^Division of Pulmonary, Critical Care, and Sleep Medicine, University of California San Diego, La Jolla, CA 92093; ^i^Centre for Innate Immunity and Infectious Diseases, Hudson Institute of Medical Research, Clayton, VIC 3168, Australia; ^j^Department of Molecular and Translational Sciences, Monash University Clayton, Clayton, VIC 3168, Australia; ^k^Sanford Burnham Prebys Medical Discovery Institute Cell & Molecular Biology of Cancer, La Jolla, CA 92037; ^l^Department of Neurology, Beth Israel Deaconess Medical Center, Harvard Medical School, Boston, MA 02115

**Keywords:** COVID-19, surfactant, lung organoids, inflammation, macropinocytosis

## Abstract

The prevalence of “long COVID” is just one of the conundrums highlighting how little we know about the lung’s response to viral infection, particularly to syndromecoronavirus-2 (SARS-CoV-2), for which the lung is the point of entry. We used an in vitro human lung system to enable a prospective, unbiased, sequential single-cell level analysis of pulmonary cell responses to infection by multiple SARS-CoV-2 strains. Starting with human induced pluripotent stem cells and emulating lung organogenesis, we generated and infected three-dimensional, multi-cell-type-containing lung organoids (LOs) and gained several unexpected insights. First, SARS-CoV-2 tropism is much broader than previously believed: Many lung cell types are infectable, if not through a canonical receptor-mediated route (e.g., via Angiotensin-converting encyme 2(ACE2)) then via a noncanonical “backdoor” route (via macropinocytosis, a form of endocytosis). Food and Drug Administration (FDA)-approved endocytosis blockers can abrogate such entry, suggesting adjunctive therapies. Regardless of the route of entry, the virus triggers a lung-autonomous, pulmonary epithelial cell–intrinsic, innate immune response involving interferons and cytokine/chemokine production in the absence of hematopoietic derivatives. The virus can spread rapidly throughout human LOs resulting in mitochondrial apoptosis mediated by the prosurvival protein Bcl-xL. This host cytopathic response to the virus may help explain persistent inflammatory signatures in a dysfunctional pulmonary environment of long COVID. The host response to the virus is, in significant part, dependent on pulmonary Surfactant Protein-B, which plays an unanticipated role in signal transduction, viral resistance, dampening of systemic inflammatory cytokine production, and minimizing apoptosis. Exogenous surfactant, in fact, can be broadly therapeutic.

## Significance

New severe acute respiratory syndrome-coronavirus-2 (SARS-CoV-2) variants continue to emerge with differing effects on the lung, yet there is a lack of readily available, biologically relevant in vitro models for studying their impact on lung epithelial cells. We have harnessed our patient-specific, induced pluripotent stem cell–derived, multicell lung organoid model to investigate critical aspects of COVID-19, including viral tropism and routes of entry, lung-specific immune response, and a role for surfactant protein B in innate immunity, viral surveillance, cell survival, and inflammation. These findings can potentially revolutionize our understanding of the lung’s intrinsic response to viral infections, leading to innovative therapies.

Our understanding of pulmonary COVID-19 caused by infection with severe acute respiratory syndrome coronavirus-2 (SARS-CoV-2) remains incomplete and ever-shifting not only because of the emergence of new viral variants but also based on our growing appreciation of the long-term persistence of symptomatology, often pulmonary (1, 2). This occurs despite what should have been adequate blockade of viral entry via canonical routes and effective pharmacologic suppression of a systemic inflammatory response (3–5). To better explore the dynamics of acute viral infection using single-cell multiomic analysis of the sequential downstream events that transpire immediately postinfection in epithelial and mesenchymal lung constituents, we used a unique in vitro system that reproduces the development, the three-dimensional (3D) cytoarchitecture, and the function of many pulmonary cell types. Starting from normal human induced pluripotent stem cells (hiPSCs), we derived 3D lung organoids (LOs) representing different cells of the lung (6–9). Because the LOs are generated from “primary” patient-derived hiPSCs, LOs could be generated from patients congenitally missing key protective factors (e.g., surfactant), as well as men and women from a number of racial and ethnic backgrounds. Furthermore, because our in vitro system allows endoderm-derived “mini-lungs” to be generated without potentially confounding contributions or influences from other organs or lineages, we could identify processes and responses that were “lung-autonomous.”

We studied the response of these LOs to acute infection with multiple variants of SARS-CoV-2. This system enabled us to appreciate a number of unrecognized and surprising aspects of viral infection which might not only lend insight into viral entry, the persistence of postinfection inflammatory cascades, and the lung’s intrinsic response to viral pathogens but may also suggest clinic-ready therapeutic strategies, based on the repurposing of at least two FDA-approved drugs.

Such unexpected findings included broader viral tropism; an intrapulmonary innate immune system generated by bystander lung cells, and a protective function against inflammation and infection by surfactant protein B (SP-B), playing an important role in signal transduction. We show that many lung cell types are infectable (10–13), not solely those bearing ACE2 and TMPRSS2, or other viral receptors. We observed a “noncanonical” (i.e., non-receptor-mediated) route of viral entry via a form of endocytosis called macropinocytosis, which can be abrogated by endocytosis blockers, including the FDA-approved drug apilimod.

We demonstrate the existence of an intrinsic, autonomously acting, intrapulmonary innate immune “first response” system initiated by the pulmonary epithelia themselves upon acute viral incursion. This system, we show, “attempts” to restore lung homeostasis by inhibiting intercellular viral dissemination, averting apoptosis, and dampening inflammatory cascades. SP-B, best known as the pivotal component of the pulmonary protein–lipid complex surfactant which decreases alveolar surface tension, helps mediate this defense mechanism by playing a critical signaling role in innate immunity, viral surveillance, and inflammation. SP-B is also secreted by airway epithelial cells, where its function had previously been poorly defined.

These roles become apparent by examining and manipulating LOs derived from hiPSCs from patients congenitally lacking SP-B, a unique capability of the experimental model we describe here. Because the absence or consumption of SP-B increases viral infectivity and dissemination, worsens cell death, and alters the endogenous inflammatory/innate immune response of pulmonary epithelial cells, we explored the therapeutic potential of administeringFDA-approved exogenous surfactant. Furthermore, we suggest that because this intrinsic antiviral defense system generates signals that beckon infiltration by more classical hematopoietic inflammatory mediators in a feedback loop fashion, optimizing this system’s initial efficacy might offer a way to preempt the postinflammatory, postapoptotic fibrosis which likely potentiates “pulmonary long COVID” (1, 2).

## Results

### hiPSC-Derived LOs Are Infectable with Multiple SARS-CoV-2 Strains, Validated in Primary Human Lung Cells.

**Fig. 1.**
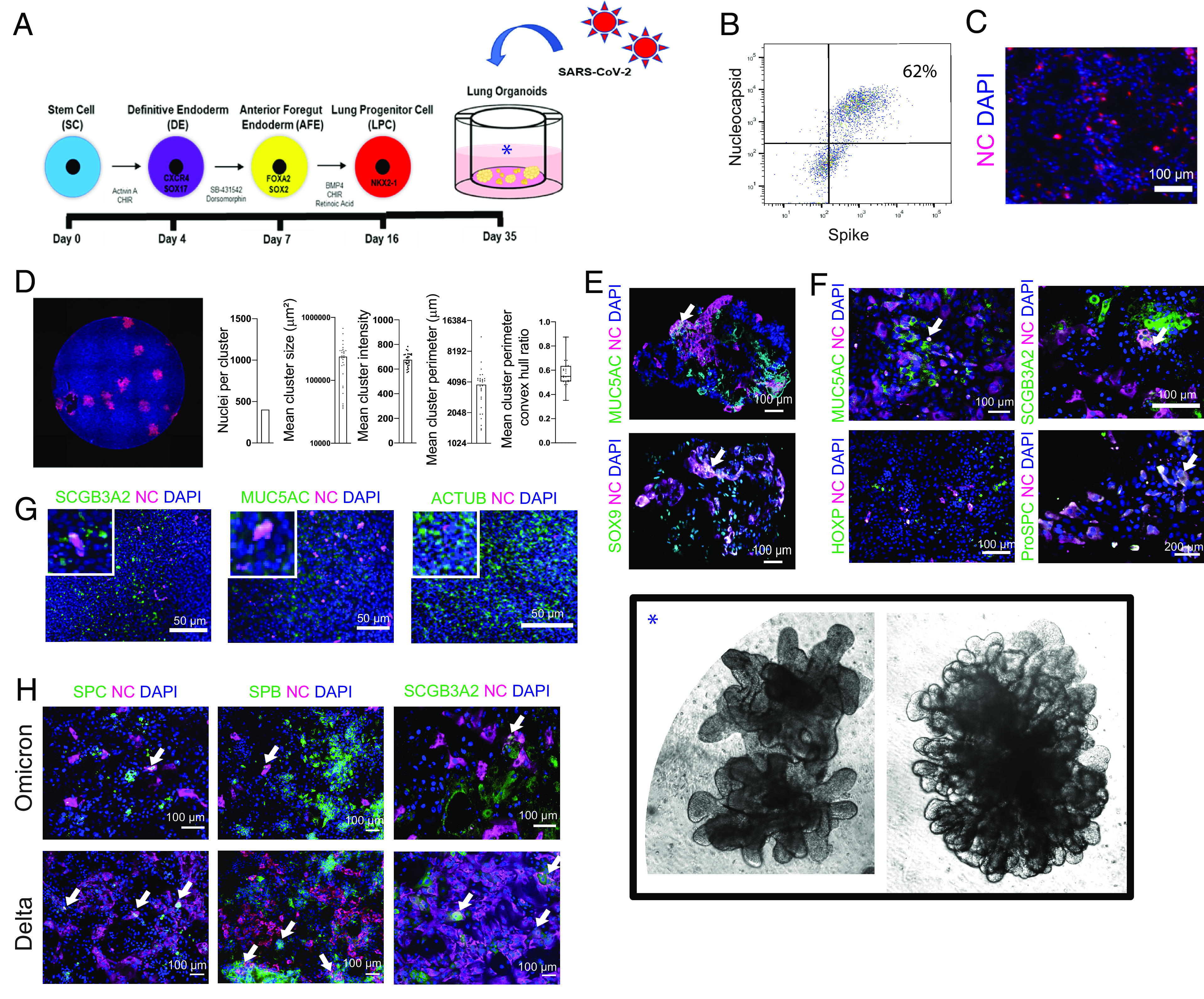
hiPSC-derived LOs are susceptible to infection with multiple strains of SARS-CoV-2, validated in primary human lung epithelia. (*A*) Schematic of steps by which hiPSCs are “instructed” to become 3D LOs that resembles a lung in situ and subsequently infected with SARS-CoV-2. See *SI Appendix,* Fig. S1, for details. Photomicrographs of actual WLOs are magnified in the *Inset* [*] in the *Lower Right* of the montage. (*B*) Representative flow cytometry of infected 3D PLOs expressing viral proteins Spike and NC confirms that ~62% of the pulmonary epithelial cells were infected. (*C*) Immunostaining of infected dissociated PLOs 24 hpi for viral NC (cyan) confirms the cytometric findings of viral infection. In all photomicrographs in this figure, a DAPI nuclear stain (blue) is used to visualize all cells in the field. (*D*) Immunostaining of infected dissociated PLOs overlayed with carboxymethylcellulose to enhance visualization and permit quantification. Cells were fixed, permeabilized, and costained with NC-AF594 antibody (red). A representative well is shown. Images were quantified with a custom-scripted Image J code. Measurements of NC^+^ nuclei/cluster, cluster size and perimeter, and cluster intensity confirm robust infection. (*E*) Immunostaining of *intact* 3D WLOs 36 hpi for coexpression of viral NC (cyan) (indicative of infection) in multiple pulmonary epithelial cell types as identified by cell type-specific immunomarkers (green). Two representative cell types are shown: goblet cells (MUC5AC) and LPCs (SOX9). Note that triple-labeled cells (NC [cyan] *plus* cell type marker [green] *plus* DAPI [blue]) typically appear white (an example of which is indicated by an arrow in this and the other Panels). (*F*) Immunostaining of dissociated 3D PLOs and DLOs with viral NC and cell-type lung epithelial markers: goblet cells (MUC5AC), club cells (SCGB3A2), alveolar type 2 cells (proSPC), and AT1 cells (HOPX). The arrow indicates representative triple-labeled cells. (*G*) To validate the range of cell types infected in LOs, primary human bronchial epithelial cell (HBEC) ALI cultures were also infected and immunostained 24 hpi. Shown in the coexpression of viral NC (evidence of infection) in a range of the same representative pulmonary epithelial cells shown above: goblet cells, club cells, and ciliated cells (AcTub). *Insets* are 2.5× original images. (*H*) Immunostaining of dissociated PLOs and DLOs 24 hpi, comparing SARS-CoV-2 variants Omicron vs. Delta. (Arrows indicate infected cells). The data are representative of at least three independent experiments. All scale bar, 100 µm unless otherwise specified.

Previous studies of SARS-CoV-2 have relied primarily on postmortem material (14–17), neoplastic cell lines (18, 19), and on cultures of selected lung constituents (13). We employed a system that enabled us to map, prospectively and in an unbiased manner, the full downstream transcriptional and functional response of human pulmonary epithelial and mesenchymal cells of the normal lung upon first exposure to SARS-CoV-2. This system of epithelial and mesenchymal lung components in a 3D configuration is schematized in Fig. 1*A* and detailed in *SI Appendix* (*Methods* and *SI Appendix*, Fig. S1) (6, 8, 9). Briefly, LOs were generated from hiPSCs by exposing them to a sequence of inductive factors which emulates normal lung organogenesis, yielding three types of LOs: those primarily composed of lung cells located in the upper airway/*proximal* lung (*PLO*), those in the alveolus/*distal* lung (*DLO*), and *whole* LOs (*WLO*) which contain cell types from the entire lung, including pulmonary neuroendocrine cells. We induced the WLOs from lung progenitor cells (LPCs) by inhibiting GSK3ß with CHIR99021 in combination with FGF7, FGF10, EGF, RA, VEGF/PGF, and finally “DCI” (“dexamethasone, *c*AMP, and isobutylxanthine”) (6, 7). Exposure to DCI yielded the final step in maturation of the WLOs within 24 h of exposure, with visible “ballooning” in response to this developmentally appropriate induction combination (*SI Appendix*, Fig. S1*B* and Movie S1). These structures contained AT2 cells (pro-SPC+) that produced surfactant, both anatomically and by protein expression (7), as well as alveolar type 1 (AT1) cells (HT1-56+), club, and basal cells (*SI Appendix,* Fig. S1*C*). Mesenchymal cells, including smooth muscle, were present which, in some LOs, conferred respiratory contraction-like motion (*SI Appendix,* Fig. S1*C* and Movie S3). By altering growth factor exposure to the LPCs, including FGF2, high dose FGF10, and DCI, we generated PLOs which contained basal (p63+), club (SCGB3A2+), and goblet (MUC5AC+) cells (9) (*SI Appendix,* Fig. S1 *B* and *D*). Passaging PLOs into an air–liquid interphase (ALI) produced functional ciliated cells (Movie S2). DLOs, derived by exposure of the LPCs to CHIR99021, SB431542, FGF7, RA, and DCI (8), had the largest number of alveolar cells (*SI Appendix,* Fig. S1 *B* and *D*).

Single-cell RNA sequencing (scRNAseq) profiling confirmed the expression of lung epithelial and mesenchymal cell markers in each of the LO types (*SI Appendix,* Fig. S2 *A* and *B*), as determined by correlating the signature genes to scRNAseq data in published datasets from fetal and adult human lung cells (20–27) and the gene annotation and analysis resource, Metascape (28). We used generated LOs from a variety of hiPSCs from men and women of different racial and ethnic backgrounds (e.g., African American, Caucasian, Hispanic) (*SI Appendix,* Table S1).

In all organoids, the presence and distribution of pulmonary cell types—as represented by the size, composition, location, proportion, and identity of Uniform Manifold Approximation and Projection (UMAP) cell clusters—were always similar from organoid-to-organoid. Such consistency and reproducibility supported the generalizability and validity of our observations. To determine the acute and primary tropism of the SARS-CoV-2 spike protein in the lung without killing the cell or permitting intercellular spread, we first infected the LOs with a replication-incompetent vesicular stomatitis virus pseudotyped with the SARS-CoV-2 spike protein conjugated to GFP (pseudovirus-GFP) (29) (*SI Appendix,* Fig. S3*A*). To confirm the cell types infected, we performed immunofluorescence staining of 3D LOs and acutely dissociated LOs in monolayer (to enhance visualization of individual cells and avoid confounding superimposition) 24 hours postinfection (hpi). We detected colocalization of pseudovirus-GFP with MUC5AC+ in the intact 3D LOs (*SI Appendix,* Fig. S3*B*). There was also colocalization of pseudovirus-GFP with cells expressing SCGB3A2 (secretory cells), and MUC5AC (goblet cells) but not with cells expressing p63 (basal cells) (*SI Appendix,* Fig. S3*C*).

Next, we infected the 3D PLOs with authentic replication-competent SARS-CoV-2 virus, variant WA1. We confirmed infection by fluorescent focus unit (FFU) titers (*SI Appendix,* Fig. S4*A*), flow cytometry using antibodies specific for the viral NC protein and spike protein (S) (Fig. 1*B*) and immunofluorescence against NC (Fig. 1*C*). Infected cells (NC**+**) were quantified with a custom-scripted Image J code (Fig. 1*D*). To determine tropism after acute infection, we stained intact and dissociated LOs for specific lung markers, as well as the viral NC. In the 3D WLOs, MUC5AC**+** (goblet) and SOX9**+** (progenitor) cells were infected (Fig. 1*E*), and, in the dissociated PLOs and DLOs, SCGB3A2**+** (secretory), MUC5AC**+** (goblet), and pro-SPC**+** (AT2) cells were infected, but not HOPX**+** (AT1) cells (Fig. 1*F*). Many of these cell types secrete surfactant proteins in the alveolus and the airway (e.g., secretory and AT2 cells—discussed in greater detail below). The tropism of the virus for these cell types in the LOs was validated in primary human bronchial epithelial cell (HBEC)-derived ALI cultures. In HBEC-derived airway epithelial cells, SARS-CoV-2 similarly infected SCGB3A2**+** (secretory), AcTub**+** (ciliated), and MUC5AC**+** (goblet) cells (Fig. 1*G*).

To explore the differing pathology of SARS-CoV-2 variants, we infected PLOs and DLOs with the Delta and Omicron variants and examined cellular targets and viral dissemination. As found above for WA1, these variants infected a similar spectrum of pulmonary cells (Fig. 1*H*). Delta disseminated in culture more efficiently in 24 h than Omicron (58% vs. 6% cells infected). Delta infected more SPC**+** and SPB**+** AT2 cells in the DLOs than Omicron, suggesting that the tropism of the Omicron variant may not be primarily in alveolar cells. This observation is consistent with clinical evidence that Delta causes a more alveolar lung pathology (acute respiratory distress syndrome, ARDS) whereas Omicron causes more upper airway inflammation.

### SARS-CoV-2 Infects Many Lung Cell Types, *Independent* of ACE2, by Exploiting a Noncanonical Endocytosis Route of Entry.

**Fig. 2.**
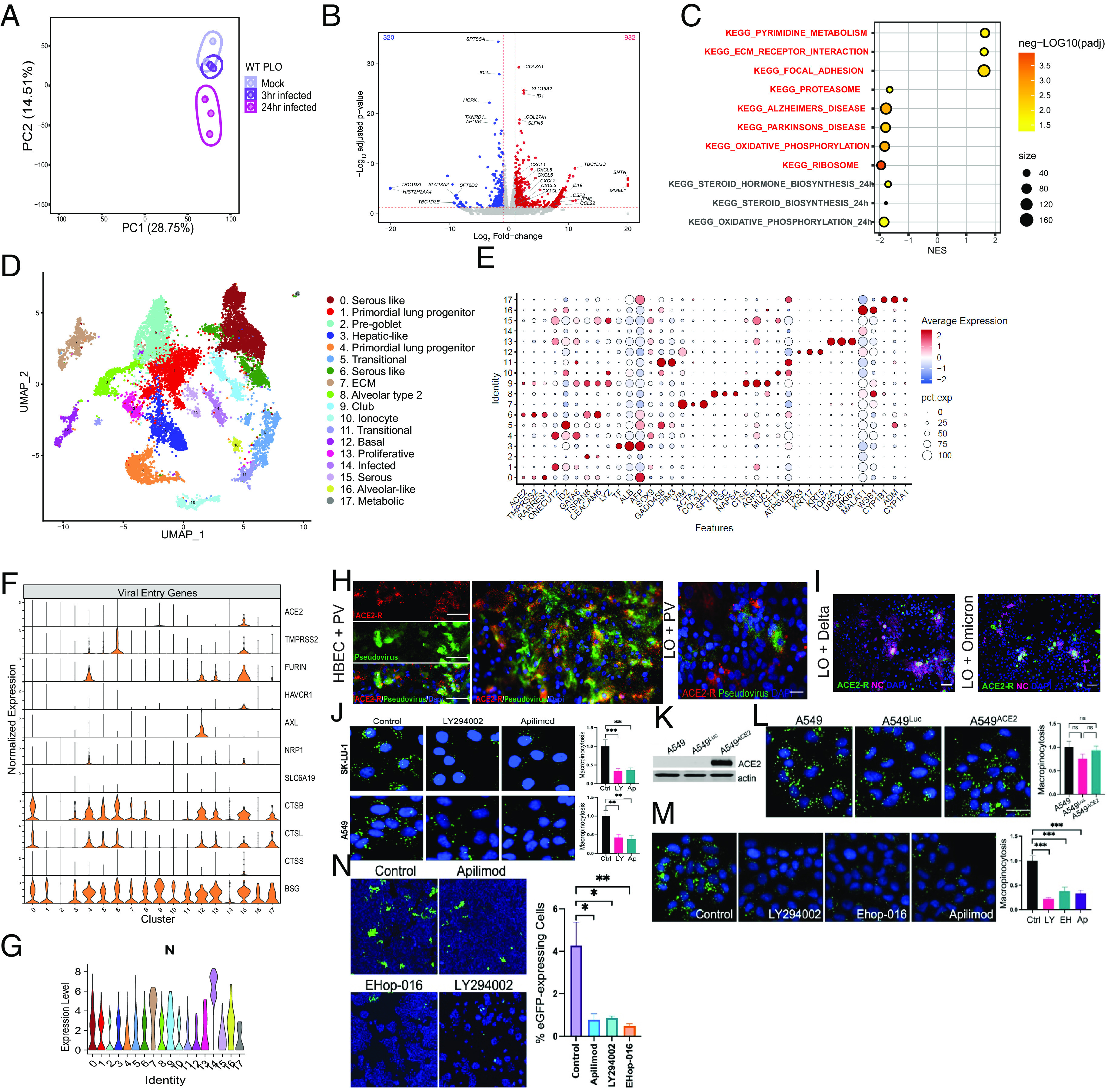
Transcriptional changes and tropism in hiPSC-LOs infected with SARS-CoV-2; many cell types can be infected, independent of ACE2. (*A*) PCA plot of the WT PLOs comparing samples that were uninfected (mock), 3 hpi, and 24 hpi. Note that PLOs infected for 24 h segregate from those infected for only 3 h or mock-infected. (*B*) Volcano plot analysis of differential expression of SARS-CoV-2 infected PLOs vs. mock infection. Note a heavy upregulation (red dots) of inflammatory cytokine. (*C*) Gene overrepresentation analysis using the KEGG pathway database of SARS-CoV-2 3 hpi vs. mock infected LOs. (*D*) UMAP of scRNAseq of 3D PLOs, 24 hpi with the alpha variant. Clusters were generated via Harmony Integration of three independent samples. The cell-type identity of each numbered, color-coded cluster is indicated in the list to the right of the UMAP. That same numbering scheme (Clusters 0 to 17) applies, as well, to Panels *E*–*G*. (*E*) Characteristic genes-per-cluster for the PLO scRNAseq data. The key to the heat map (degree of expression) and size of the circles (percent expression) is indicated toward the *Right* of the panel. (*F*) Violin plots of SARS-CoV-2 entry genes (including ACE2) present in each cluster in the PLOs. (Cluster identification as per *E*). (*G*) Violin plot of the SARS-CoV-2 NC transcript in the same clusters shown in Panel *F*. In cross-referencing between the two panels, note that N is present in some cells that contain the ACE2 transcript (e.g., Cluster 15), but also, significantly, in clusters that do not (e.g., Cluster 12). Viral genes in addition to N (further indicative of infection) are shown in these same clusters (and in every cluster) in *SI Appendix*, Fig. S4*C*. (*H*) Immunofluorescence images of both primary HBECs (*Left*) and acutely dissociated PLOs (*Right*) infected with SARS-CoV-2 pseudovirus show the pseudovirus in cells with and without ACE2, 4.1 ± 1% of the former and 0.72 ± 0.2% of the latter (*I*) Immunofluorescence images of dissociated hiPSC-PLOs infected with Delta and Omicron. Show viral NC in cells with and without the ACE2 receptor. (*J*–*N*) Proof that SARS-CoV-2 employs macropinocytosis (an endocytotic process quantified by FITC-Dextran [green] uptake) to enter lung cells, independent of ACE2, but which apilimod can block. (*J*) Prior to infection, SK-LU-1 lung cells were treated with 25 µM LY294002 (PI3K inhibitor) and A549 lung cells were treated with 100 µM LY294002, both used to block macropinocytotic mechanisms. Apilimod’s effects were then compared against these treatments. Macropinocytosis (green fluorescence) was inhibited by all drugs comparably; DMSO served as the negative control; **P<0.01 and ***P<0.001. (*J*) To show that ACE2 does not affect the extent of macropinocytosis, A549 cells were engineered to express ACE2 or just luciferase (Luc). Actin served as a loading control in the western blot. (*L*) FITC-Dextran uptake (macropinocytosis) was similar in A549 WT, A549^Luc^, and A549^ACE2^ cells (ns = not significant). (*M*) A549^ACE2^ cells were treated with LY294002, Ehop-016 (another endocytosis inhibitor via its suppression of Rac1 which perturbs actin cytoskeleton dynamics, and hence another positive control), apilimod, or DMSO (control). Macropinocytosis was still inhibited by all compounds, ***P < 0.001. (*N*) All the macropinocytosis inhibitors in *L* blocked SARS-CoV-2 infection of A549 cells as quantified by a large reduction in pseudovirus-eGFP-positivity (green) in those pretreated cells, compared to DMSO pretreatment. At least 15,000 cells/condition were analyzed for n = 3 replicates. *P < 0.05 and **P < 0.01.

To determine the response of pulmonary cells to infection at different timepoints after infection, we performed unbiased genome-wide transcriptome profiling on PLOs at 0 (mock), 3, and 24 hpi. We identified 637 differentially expressed genes (DEGs) between 0 to 3 hpi, and 1,302 DEGs between 0 to 24 hpi. Principal component analysis (PCA) showed that PLOs at 24 hpi occupied a distinct transcriptional space compared to mock-infected PLOs (Fig. 2*A*). Volcano plots of the mock-infected vs. 24 hpi PLOs demonstrated induction of multiple chemokine and cytokine transcripts including *CCL22, CXCL-1, -2, -3, -5,* and *-6, IFNE,* and *CSF3* [the latter being the most up-regulated in infected HBECs postinfection (30)] (Fig. 2*B*). Interestingly, *HOPX*, a gene associated with alveolar type 1 (AT1) cells (31, 32), was down-regulated despite AT1 cells not being primarily infected (Fig. 1*F*). *TXNRD1*, an enzyme important in protecting cells against oxidative stress (33), was also decreased. Collagen genes, including *COL3A1* and *COL27A1*, were increased, suggesting an early fibrotic phenotype.

Gene set enrichment analysis (GSEA), using Kyoto Encyclopedia of Genes and Genomes (KEGG), of the mock vs. 3 hpi PLOs, revealed enrichment of pathways associated with pyrimidine metabolism and extracellular matrix (ECM) receptor interaction (Fig. 2*C*), pathways previously associated with pulmonary infection (34, 35). Mock vs. 24 hpi PLOs revealed enrichment pathways associated with oxidative phosphorylation (36–38) and steroid biosynthesis. Ingenuity pathway analysis (IPA) revealed associations with IL-17 signaling, pulmonary fibrosis idiopathic signaling, and IL-8 signaling (*SI Appendix,* Fig. S4*B*).

These data emphasize that normal lung epithelial cells, without the intercession of hematopoietic-derived cells or a circulatory system, can initiate innate immune responses to SARS-CoV-2 infection, impact gene expression related to stress resistance, and influence epithelial to mesenchymal transitioning at this mucosal barrier (39–41).

To determine the response of individual pulmonary cells to SARS-CoV-2, we performed scRNAseq 16 hpi in 3D PLOs (Fig. 2*D*). First, we identified infected cell clusters based on expression of viral gene transcripts (*SI Appendix,* Figs. S2*G* and S4*C*). As shown in Fig. 2*G*, the SARS-CoV-2 nucleocapsid (NC) transcript (*N*), was detected in most clusters at various expression levels. Cluster 14 had the highest expression of all the viral genes but was not representative of a specific cell type or function (Fig. 2*E*). This cluster may have represented a mix of infected cells in the process of dying, with similar gene expression profiles. Cluster 7 (ECM) had the next highest expression of the viral genes, and clusters 9 (club cells) and 16 (alveolar-like cells) had the third highest (Fig. 2*E*). As noted above, some of these cells are surfactant secreting, a point that will be of interest below.

In infected clusters, we next correlated the expression of the above-mentioned viral genes with genes encoding the primary mediators of SARS-CoV-2 entry, *ACE2* and *TMPRSS2* (42) (Fig. 2*F*). As might be expected, the virus’ NC transcript was coexpressed in cells that also contained an ACE2 transcript (cross-reference Fig. 2*G* with Fig. 2*F*) (e.g., Cluster 15). However, the NC transcript was also found in pulmonary cell types that do *not* to bear ACE2 (e.g., Cluster 12). (*SI Appendix*, Fig. S4*C* shows that viral genes in addition to N—further indicative of infection—are present in every cluster).

Interestingly, ECM, alveolar-like cells, and the infected cluster showed the *highest* expression of viral genes but the *lowest* expression of *ACE2* and *TMPRSS2*. We then interrogated other potential viral entry genes (43), dividing them into *“canonical”* genes (which, in addition to *ACE2* and *TMPRSS2*, may include receptors such as *FURIN, HAVCR1, AXL, NRP1, SLC6A19,* and *BSG* [CD147]), and *noncanonical* entry routes (such as the genes for the cathepsin proteins *CTSB, CTSL,* and *CTSS*) (Fig. 2*F*). *CTSB* and *CTSL* encode proteins pivotal for *endocytosis* (44–46). BSG is a constitutively expressed surface glycoprotein receptor in epithelial cells (47), but has a unique relationship with the spike protein. We found *BSG* expressed in 16 of the 18 clusters and *CTSB* expressed in 11 of the 18 clusters. Serous and mucous-like cells expressed the anticipated viral entry genes, *ACE2* and *TMPRSS2*, as well as *CTSB* and *CTSL*. Primordial lung progenitor, pregoblet, club, and ionocytes expressed only *BSG*.

We verified the scRNAseq findings regarding SARS-CoV-2 tropism using immunofluorescence. Acutely dissociated LOs and HBEC-ALI were examined 24 hpi with pseudovirus-GFP. Infected cells coexpressed ACE2 and GFP from the pseudovirus (4.1 ± 1%), yet there were also cells that expressed *only* pseudovirus-GFP (0.72 ± 0.2%) (Fig. 2*H*). Dissociated LOs infected with the replication-competent live Delta and Omicron showed similar tropism, with some cells coexpressing ACE2 and viral NC, but others expressing *only* NC (Fig. 2*I*).

Guided by our gene and protein expression data, we suspected that the non-ACE2-mediated infection we were observing was via a noncanonical route, such as an endocytic mechanism that can function independently of ACE2. One such mechanism is macropinocytosis, an uptake pathway that is nonselective and functions in bulk fluid-phase endocytosis. To interrogate the feasibility of such a noncanonical route mediating SARS-CoV-2 entry into lung cells (Fig. 2 *J*–*N*), we used compounds known to block macropinocytosis. Macropinocytotic uptake is regulated by PI3K signaling (which plays a critical role in macropinosome closure and fission from the plasma membrane). Therefore, we used the PI3K inhibitor LY294002, as well as Ehop-016, an inhibitor of Rac1 that perturbs actin cytoskeleton dynamics. Phosphatidylinositol-3-phosphate 5-kinase (PIKfyve) functions to distribute endocytic cargo and has been shown to suppress macropinosome maturation (48). We hypothesized that blocking PIKfyve activity could be particularly selective in this context for also blocking the early stages of macropinocytosis that facilitate cargo capturing from the extracellular space. Therefore, we also used apilimod, an FDA-approved PIKfyve inhibitor, to block macropinocytosis, and determine whether this has an effect on infection (49). Apilimod blocked the uptake of high molecular weight FITC-Dextran, a marker of macropinosomes, in A549 and SK-LU-1 lung cells as efficiently as LY294002 and Ehop-016 (Fig. 2 *H* and *M*). ACE2 expression in A549 lung cells did not affect the extent of macropinocytosis (Fig. 2 *K* and *L*) nor macropinocytosis blockade by apilimod, LY294002, or Ehop-016 (Fig. 2*M*). Having validated apilimod as a tool for inhibiting macropinocytosis, the drug was then applied to A549 cells, engineered with a lentivirus transducing ACE2, and exposed to pseudovirus-eGFP. All cells were infectable and all endocytosis-inhibiting compounds significantly reduced the number of infected (eGFP**+**) cells including apilimod (Fig. 2*N*). The efficiency with which apilimod blocked infection suggests that SARS-CoV-2 does enter pulmonary epithelial cells by noncanonical endocytotic as well as canonical receptor-mediated routes. Contemporaneous with our findings in lung cells, Rac1-dependent macropinocytosis was shown to enable SARS-CoV-2 entry also into kidney cells (50).

We compared the efficiency of reducing viral infection (in hiPSC-derived PLOs from diverse donors) using a macropinocytosis-blocking agent (apilimod) vs. a cathepsin-blocking agent (ONO5334) (51) [with a viral replication-blocker, remdesivir (52), as a positive control]. At 24 hpi, apilimod reduced SARS-CoV-2 infection more than ONO5334, but less than remdesivir (*SI Appendix,* Fig. S5 *A* and *B*).

These data suggest that repurposing the FDA-approved drug apilimod may be a synergistic therapeutic option early in SARS-CoV-2 infection to block alternative routes of entry by the virus when it circumvents ACE2/TMPRSS2-mediated routes. The failure of the cathepsin inhibitor ONO5334 to block infection suggests that the cathepsins may not be an ideal therapeutic target.

### Acute Infection with SARS-CoV-2 Induces an Autonomous Intrapulmonary Inflammatory Cascade.

**Fig. 3.**
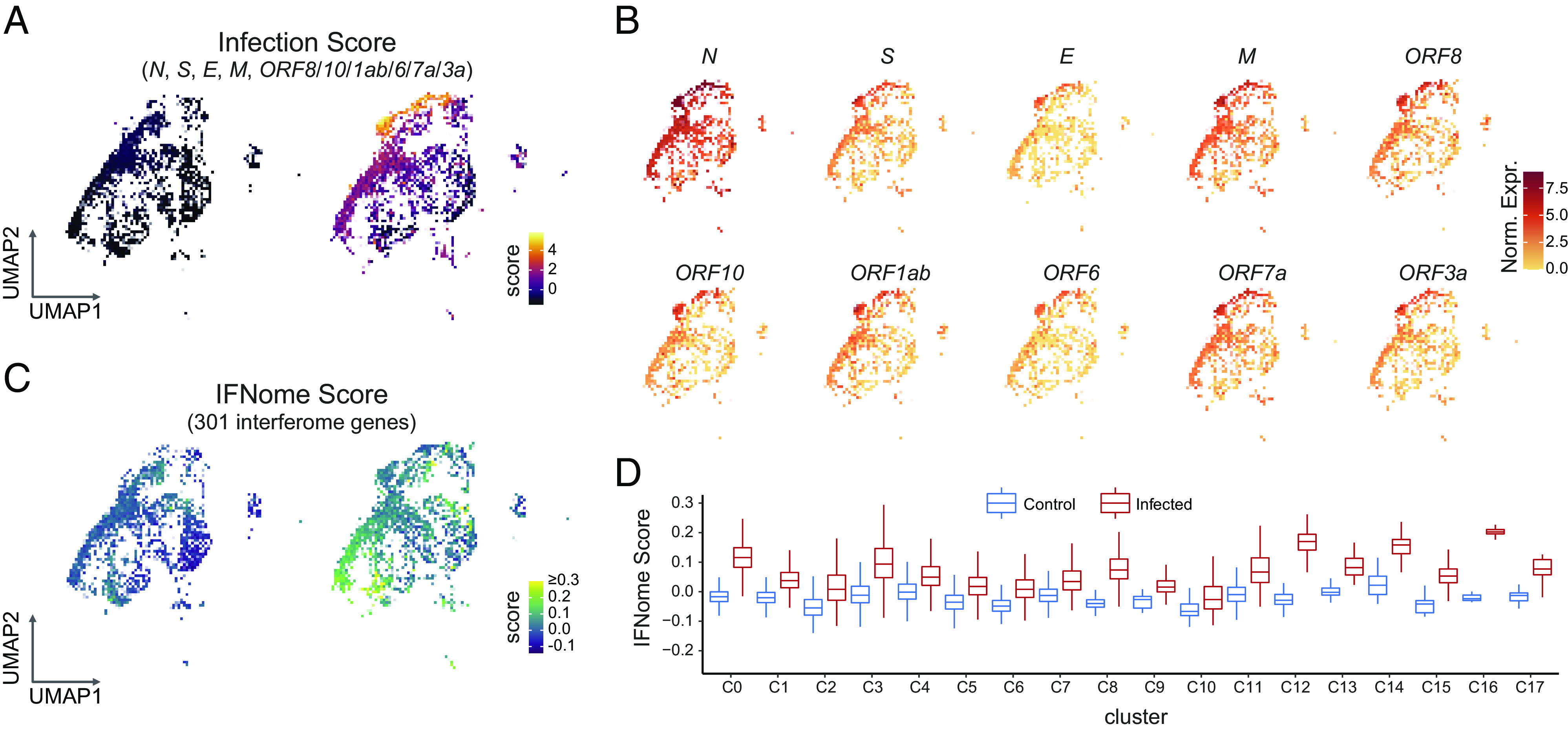
Acute infection with SARS-CoV-2 induces a lung-autonomous intrapulmonary inflammatory cascade in bystander cells. (*A*) UMAP of infection score showing the highest expression of SARS-CoV-2 genes in clusters 5 and 9. The significance of these clusters is described in the text. Highest expression levels are in yellow. (*B*) UMAP of viral gene expression. While *N* (viral NC) is expressed in all clusters, the complete complement of viral genes is expressed in clusters 5 and 9. Highest expression levels are in red. (*C*) UMAP depicting interferome-I (IFN-I) score for each cluster. Highest expression is seen in clusters 0, and 12 to 17. Clusters with the highest IFN-I scores did not overlap with clusters having the highest infection scores, suggesting that noninfected “bystander” lung cells were being induced by infection of their lung cell “neighbors” to mount an IFN response. Highest expression levels are in yellow. (*D*) Bar plots depicting a higher IFN-I score for each cluster when comparing mock vs. infected LOs.

To determine the relationship between viral infection and the intrinsic epithelial cell response to such, we analyzed the scRNAseq data after infecting a mix of all the LO types (PLO, DLO, and WLO) (*SI Appendix,* Fig. S6 *A* and *B*). We scored the clusters based on the expression of the viral genes *N, M,* and *ORF10.* Clusters 5 (alveolar progenitor cells) and 9 (infected, “nonspecified pulmonary” cells) showed the highest infection score (Fig. 3*A*). When looked at separately, the viral gene *N* was highly expressed throughout all the clusters, followed by *M, ORF8*, and *ORF7a* (Fig. 3*B*). We then assessed the expression of type I interferon-inducible genes using an open-access interferome database generated from gene expression profiles of cells treated with type I or II interferon (52, 53). We generated an *interferome score* for each cluster, with significant elevations of interferome scores in all clusters (Fig. 3 *C* and *D*). Analysis showed that absolute levels of viral transcript did not directly correlate with abundance of interferome gene transcripts. We then looked at specific examples of interferome gene expression profiles and found increased expression in *IFI27, IFI6, IFIH1, IFIT-1, -2, and -3,* and *IFITM1* (*SI Appendix*, Figs. S7 *A* and *B*). Chemokines were also highly expressed in most clusters except for the highly infected cluster 5 which constitutively expressed *CXCL1, CXCL2, CXCL3*, and *CCL20* at high levels (*SI Appendix*, Fig. 7*C*). The distribution of chemokine expression did not correlate with either interferome gene expression or viral gene expression. Finally, we performed a canonical pathway analysis and interrogated the disease and biological functions and upstream regulators using IPA (54). The Coronavirus Pathogenesis pathway had positive activation z-scores in most of the clusters, while EIF2 signaling had negative activation z-scores (55) (*SI Appendix*, Fig. S7*D*). Genes important in apoptosis and necrosis were increased (*SI Appendix*, Fig. S7*E*), as were the upstream regulators of these canonical pathways *IFNL1, IFNA2, NONO, IRF7,* and interferon alpha (*SI Appendix*, Fig. S7*F*). The correlation of viral gene expression with these inflammatory mediators again showed nonoverlapping profiles.

Taken together, these data suggest that specialized lung cells are equipped to respond to infection with different innate immune mechanisms. These data further suggest a threshold for viral expression that triggers differential expression of the interferome and chemokines in human lung cells *without* the intercession, or even presence, of hematopoietic derivatives or a vascular compartment.

### The Unexpected Role of Pulmonary Surfactant in the Intrinsic Intrapulmonary Inflammatory Defense and Cell Death.

Having demonstrated the surprisingly broad susceptibility of human lung cells to SARS-CoV-2 through both canonical and noncanonical viral entry routes, and the intrinsic ability of normal pulmonary epithelia themselves to promote innate immune responses, we next turned to the molecular mediators of these downstream events—after viral entry, by whatever mechanism. A hint in this regard came, surprisingly, from the actual clinical presentation of pulmonary COVID-19, offering clues as to what unique intrapulmonary mechanisms might be involved. The ARDS caused by this virus can be atypical (56). Based on autopsy material from patients who have succumbed to COVID-19-related ARDS, there is a reduction in SP gene expression in the lung (14, 15). There are four types of SPs: A, B, C, and D. SP-B is the most critical component of pulmonary surfactant, a proteolipid complex secreted primarily by alveolar type 2 (AT2) cells to reduce surface tension in the alveoli, thereby facilitating gas exchange. Club cells make different isoforms of SP-B for different functions. SP-C also contributes to lowering surface tension, SP-A and SP-D collaborate with SP-B to maintain the airway epithelial lining fluid, and SP-A and SP-D perform innate immune functions (57, 58).

During human fetal development, SP-B is gestationally regulated (59, 60). A developmental deficiency of surfactant is the most common cause of neonatal RDS in preterm newborns (61), characterized by poor pulmonary compliance and refractory hypoxemia (62, 63). ARDS associated with COVID-19 is clinically similar in some instances (64, 65). We were prompted, therefore, to focus next on the role of surfactants during viral infection.

**Fig. 4.**
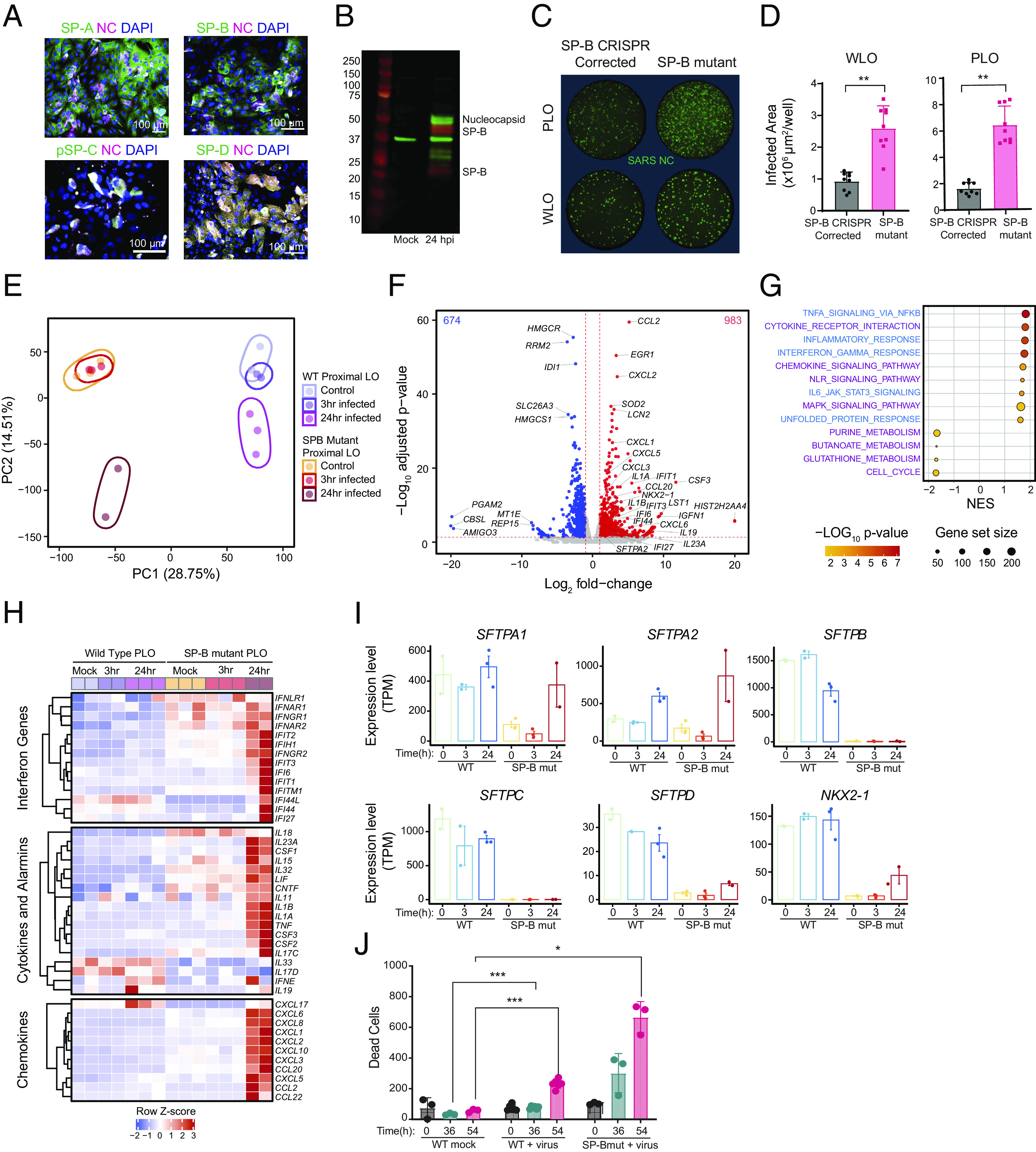
Surfactant suppresses viral infection and modulates inflammation. (*A*) Immunostaining of acutely dissociated PLOs 24 hpi, showing expression of SPs A, B, C, and D in many lung cell types. (*B*) Western blot of mock and PLOs 24 hpi. Infected organoids show viral NC expression as well as 2 isoforms of SP-B—the proform (25 kD) and the pre–pro form (42 kD). (*C*) Immunostaining for NC in LOs from an SP-B deficient patient (PRO133) and in the same LOs corrected via CRISPR genomic editing to enable SP-B production. The absence of SP-B enabled greater infection of these isogenic LOs than when SP-B production was restored. (*D*) Data from this experiment, which was repeated nine times, are graphed as total infected cell area. ****P < 0.0001. (*E*) PCA plot of WT PLOs following mock infection (lavender), 3 hpi (purple), 24 hpi (pink) vs. SP-B-deficient PLOs following mock infection (orange), 3 hpi (red), 24 hpi (brown). Note that infected LOs lacking SP-B segregate dramatically from those expressing SP-B and that 24 hpi in the absence of SP-B creates another distinct cluster (see text for details and explanation). (*F*) Volcano plot of differential gene expression of SARS-CoV-2 infected SP-B-deficient PLOs vs. mock infection. The red lines indicate a *P* < 0.05. The absence of SP-B is associated with upregulation of numerous toxic cytokines. (*G*) Gene overrepresentation analysis (using KEGG pathway database) of viral- vs. mock-infected SP-B-deficient PLOs (24 hpi). (*H*) Heat map showing average expression (z-score) of key innate and inflammatory genes in WT and SP-B-deficient PLOs at 0, 3, and 24 hpi. Inflammatory pathways are heavily up-regulated in the latter compared to the former. The individual numerical value-per-condition is listed in *SI Appendix,* Table S1. (*I*) Surfactant proteins and key transcription factor *NKX2-1* expression in WT and SP-B-deficient PLOs at 0, 3, and 24 hpi. Note that the expression of *SFTPA1* and *SFTPA2* increase in both WT and SP-B-deficient PLOs, 24 hpi. The data are representative of 3 independent experiments. (*J*) SP-B deficiency impairs cell viability within LO cells following SARS-CoV-2 infection compared to WT LOs. Both types of LOs were infected with SARS-CoV-2 at MOI=0.1, and propidium iodide (PI, a marker of cell death) intake was tracked over 54 h. Significance calculated using 2-way ANOVA. *P < 0.05, ***P < 0.001, and ****P < 0.0001. SP-A, -B, -C, and -D = Surfactant Proteins A, B, C, and D, respectively; NC = viral nucleocapsid; WLO = whole lung organoid; PLO = proximal lung organoid; MUT = deletion mutation producing SP-B deficiency.

To understand the effect of surfactant on the susceptibility of a cell to SARS-CoV-2 infection, we first used immunofluorescence to probe for the four SPs 24 hpi in the WLOs and DLOs. We detected coexpression of viral NC in SP-A**+**, SP-B**+**, SP-C**+**, and SP-D**+** cells (Fig. 4*A*). Intriguingly, only infected cells expressed SP-C and SP-D while both infected and uninfected bystander cells expressed SP-A and SP-B. SP-B drew our greatest attention, because its presence (complexed with the lipid DPPC), is imperative for pulmonary surfactant function. It is required for normal lamellar body biogenesis (66–68) and regulates SP-C posttranslational modification (69). The RDS seen in preterm newborns can be temporarily improved by administering exogenous surfactant until the baby’s own synthesis ensues. In rare cases, mutations in the SP-B gene preclude SP-B ever being produced, precipitating a lethal respiratory failure reversed only by lung transplantation (70). hiPSCs generated from such neonates provided us with ideal LOs for rigorously testing SP-B’s role in viral infection.

As noted above, SP-B is produced in response to infection. On western blots of PLOs 24 hpi, we detected the 2 isoforms of SP-B secreted by club cells, 42 kDa and 25 kDa (60) (Fig. 4*B*). AT2 cells produced the 8kDa SP-B isoform used in vivo to reduce surface tension (71). The induction of SP-B postinfection in pulmonary cells, including those that do not produce surfactants in vivo to facilitate ventilation, suggested that SP-B may here be playing an antiviral, homeostatic, and/or anti-inflammatory role, at least in the upper airway (72–74).

To explore SP-B’s roles most rigorously, we not only included normal hiPSC-derived LOs but also LOs generated from hiPSCs from a patient with the above-described lethal mutation in the gene encoding SP-B, *SFTPB p.Pro133GlnfsTer95* (PRO133) (75, 76). This mutation is a homozygous, loss-of-function mutation that results in a frameshift leading to an early stop codon. The transcript is unstable and thus does not translate a protein product (7). We also generated LOs from these same hiPSCs in which this mutation was corrected via CRISPR-mediated genome editing (77). PLOs and WLOs generated from both lines were infected and NC immunoreactivity was quantified (Fig. 4*C*). SP-B-mutant LOs (PRO133) had higher numbers of infected (NC**+**) cells compared to the isogenic CRISPR-corrected cells (Fig. 4*D*). These data suggested that SP-B reduces SARS-CoV-2 infection.

To investigate how SP-B may be impacting viral entry and subsequent dissemination to neighboring cells, we compared SP-B-mutant PLOs to normal PLOs by bulk RNA-sequencing at 0, 3, and 24 hpi with SARS-CoV-2. Strikingly, PCA demonstrated that SP-B-mutant PLOs at 24 hpi occupied a distinct transcriptional space from all the wild type (WT) samples as well as the mock and 3 hpi SP-B mutant samples (Fig. 4*E*). We determined the proportion of cells infected by examining the viral reads 24 hpi and found that 16-20% of the SP-B mutant PLOs were infected compared with <1% of normal PLOs (*SI Appendix,* Table S2). Although gene expression was strikingly different between the SP-B mutant and WT LOs, characterization of the cell types using scRNAseq showed a similar ratio of clusters in all PLOs (*SI Appendix*, Fig. S8*A*).

Infected SP-B-mutant PLOs were characterized by increased chemokine transcripts (*CCL20, CXCL-1, -2, -3, -5,* and *-6*), interleukin transcripts (*IL1A, IL1B, IL19,* and *IL23A*), interferon-inducible genes, and other cytokines including *CSF3* (Fig. 4*F*). The surfactant-associated genes *SFTPA2,* and *NKX2-1* were also significantly induced, in contrast to the normal PLOs. GSEA, using KEGG, of the mock vs. 24 hpi SP-B-mutant PLOs showed activation of pathways associated with Cytokine Receptors, Chemokine Signaling, NOD-like receptor signaling, Interferon signaling, and MAPK Signaling (Fig. 4*G*). IPA was consistent with the WT PLO data except that the average expression of the canonical pathways (*SI Appendix,* Fig. S4*B*) and upstream regulators was greater in the SP-B mutant PLOs. Even the IFNome 90 enrichment scores were higher in the SP-B mutant PLOs compared to the WT PLOs (*SI Appendix,* Fig. S4*D*).

Although the uninfected SP-B-mutant PLOs had increased expression of some interferon-inducible genes, we found significant differences in the basal levels of the key protective interferon-inducible genes *IFI44L* and *IF127,* which were higher in the normal PLOs compared to the SP-B mutant PLOs (Fig. 4*H*). This finding reinforced our speculation that SP-B plays a homeostatic role in maintaining lung function by modulating inflammatory gene expression at steady state and not simply as a physical–chemical barrier. Also, mediators that correlate with *severity* of SARS-CoV-2 infection - *IL1A, IL1B, CCL2, CXCL8, CXCL10*, and *CSF3* - were induced in SP-B mutant PLOs 24 hpi (78).

To investigate further the role of SP-B in gene regulation, we measured the expression of SP genes and their transcription factor *NKX2-1*. The normal PLOs had higher expression of all the surfactant protein genes and *NKX2-1* pre- and postinfection compared to the SP-B-mutant PLOs, except for *SFTPA2*, which had the highest expression 24 hpi in SP-B-mutant PLOs (Fig. 4*I*).

To determine viral tropism in the SP-B mutant PLOs, we performed scRNAseq 16 hpi (*SI Appendix*, Fig. S8). The cell cluster types were comparable to the WT PLOs (*SI Appendix*, Fig. S8*A*). SARS-CoV-2 transcripts were detected in most clusters at various expression levels, notably cluster 14, which had the highest expression of all the viral genes but was not representative of a specific cell type or function, consistent with findings in the infected WT PLOs (*SI Appendix*, Fig. S8*B*). In the SP-B mutant dataset, alveolar cells had even more viral transcripts compared to the WT PLOs. The viral entry receptors differed between the WT and SP-B mutant data: *ACE2* and *TMPRSS2* were limited to 1 to 2 clusters in the infected WT PLOs (Fig. 2*F*), but, in SP-B mutant cells, they were highly expressed in lung progenitors, AT2 cells, club cells, and ionocytes. The other viral entry genes were consistent between the WT and SP-B mutant PLOs except with club cells expressing more transcripts of *CTSS, CTSL*, and *CTSB* (*SI Appendix*, Fig. S8*C*).

Taken together, these data suggest that the *absence* of SP-B abets greater viral infection, suppresses baseline antiviral defense mechanisms, and enables a more inimical postviral inflammatory response.

### Surfactant Reduces SARS-CoV-2 Infection and Prevents Apoptotic Death.

To evaluate whether altered viral gene expression in SP-B mutant PLOs also affected cell viability, we tracked cell death over 90 h and found that the SP-B-deficient PLOs showed higher rates of cell death after 36 h, compared to WT PLOs (Fig. 4*J*).

**Fig. 5.**
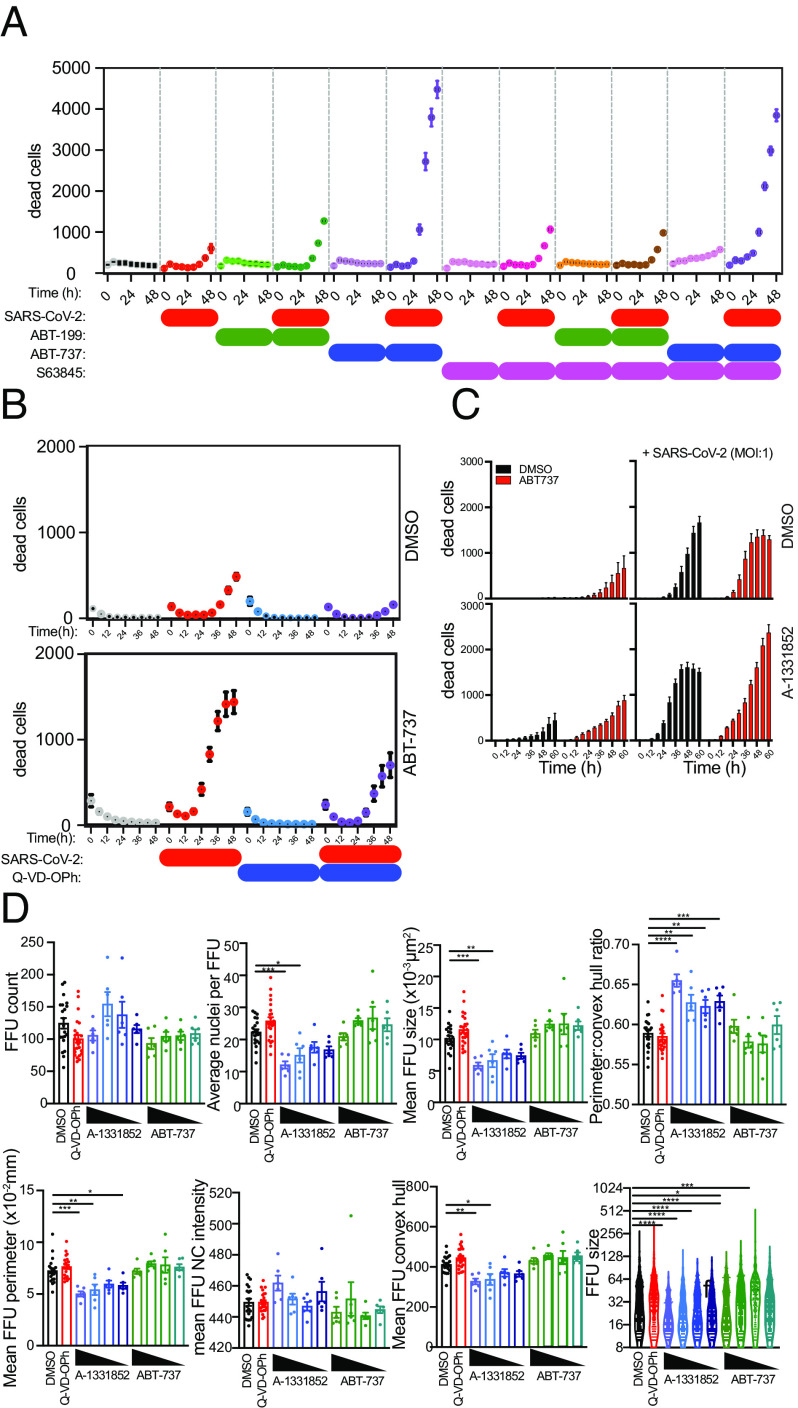
Human lung cell viability in LOs following SARS-CoV-2 infection is reduced by BH3 mimetics but increased by caspase antagonists. See *SI Appendix*, Fig. S8 for live cell phase contrast imaging of LOs showing morphological changes triggered by viral vs. mock infection (showing daily progressive dissolution of integrity of the LOs. The FFU assay is also illustrated there. (*A*–*D*) SARS-CoV-2 infection of human lung cells reduces their viability. Cell death was tracked using PI uptake. Cells were infected with SARS-CoV-2 and then treated with *ABT-737* (an inhibitor of prosurvival Bcl-2 family proteins Bcl-2, Bcl-x_L_, and Bcl-w), *ABT-199* (which modestly activates proapoptotic Bcl-2 family proteins), *S63845* (an antagonist of Mcl-1), or *Q-VD-OPh* (a caspase inhibitor). Viability was monitored every 6 h. Data from three independent experiments are shown. (*B*) Q-VD-OPh blocked virus-induced death, consistent with a role for apoptotic caspases. (*C* and *D*) ABT-7 and ABT-199 accelerated cell death (the former more than the latter), consistent with a role for Bcl-2 family proteins in regulating the dynamics of cell viability during infection.

We then evaluated the type of cell death that SP-B was ameliorating. We have previously shown that SARS-CoV-2 infection induced endoplasmic reticulum stress and an unfolded protein response, triggering caspase-mediated apoptosis (79). This process has been reported in postmortem lung tissue from COVID-19 patients and in SARS-CoV-2-infected Vero E6 cells. To understand the role of virus-triggered apoptosis in human lung cells, we infected PLOs with SARS-CoV-2 and then tracked the changes in viability in cells treated with caspase inhibitors, Bcl-2 inhibitors, and BH3 mimetics(Figs. 5 *A* and *B* and *SI Appendix*, S9). Caspase inhibition with Q-VD-OPh blocked SARS-CoV-2-induced death of human lung cells, consistent with a role for apoptotic caspases in their death (Fig. 5*B*). Inhibition of prosurvival Bcl2 family proteins Bcl-2, Bcl-x_L_ (“BCL-2–like 1”), and Bcl-w with ABT-737 or, importantly, Bcl-x_L_ alone (with A-1331852) (Fig. 5*C*) induced apoptosis of lung cells, which was enhanced by viral infection, consistent with a role for Bcl-2 family proteins in regulating cell viability during SARS-CoV-2 infection. Inhibition of Bcl-2 selectively with ABT-199 or of Mcl-1 selectively with S63845 caused minimal changes in cell viability. Taken together, these data confirmed a vital role for Bcl-2 family proteins (particularly Bcl-x_L_) in controlling the kinetics of human lung cell apoptosis triggered by SARS-CoV-2.

The death of a virus-infected cell serves as an endpoint for virus replication within that cell, but it is not known whether apoptosis prevents viral dissemination to surrounding cells. Virus infection results in transcriptional changes in cell death regulators of human lung cells, including Bcl-2 family proteins, suggesting that regulated cell death may serve to restrict viral dissemination. We hypothesized that rapid induction of apoptosis using BH3 mimetics would restrict virus dissemination by selectively targeting infected cells primed for apoptotic death. We developed a quantitative FFU assay to record the size of foci and the number of infected cells within each focus. Using a custom-scripted Image J code for automated image analysis, and a custom R Shiny tool called FFUTrackR for data visualization of the analysis output, we assessed characteristics of fluorescent foci in virus-infected cultures (Fig. 1*D*).

Treatment of virus-infected cultures with the Bcl-x_L_ antagonist A-1331852 resulted in a reduction in size of foci of infection as assessed by the number of infected nuclei per cluster, the FFU cluster area, the FFU cluster perimeter, and the FFU cluster convex hull. Inhibition of Bcl-x_L_ did not change the number of foci or viral NC levels within the FFU cluster as assessed by the average fluorescence intensity of intracellular SARS-CoV-2 NC (*SI Appendix,* Fig. S9*B*). These data suggested that rapid induction of apoptosis of virus-infected cells can occur at an early phase of the viral life cycle that interferes with virus replication and dissemination. In contrast, treatment with Q-VD-OPh did not alter virus dissemination, suggesting that apoptotic caspases do not inactivate virus, nor are they required for virus dissemination (*SI Appendix,* Fig. S9*B*). These data indicated that induction of apoptosis fails to prevent virus dissemination and releases preformed virions. Indeed, the spread of viruses from dying cells to neighboring cells is the basis for the plaque assay. These data indicated that apoptosis could restrict viral dissemination, but only if it is engaged before significant viral replication occurs in the cell. In other words, these data suggest that rapid induction of apoptosis within infected lung cells at an early phase of the viral life cycle induces the lung cells inherently to interfere with further virus replication and dissemination, an intrinsic self-defense mechanism.

### SP-B Mutant Gene Correction, Recombinant SP-B (rSP-B), and Exogenous Surfactant All Reduce SARS-CoV-2 Infection.

We have shown that LOs lacking SP-B, exhibit increased rates of SARS-CoV-2 infection and apoptotic cell death, presumably because surfactant not only provides a barrier to infection but also mediates a first-line acute intrapulmonary inflammatory host defense that combats virus survival and dissemination. This speculation was supported by our observation that CRISPR-mediated correction of a mutation in the gene encoding SP-B reversed these processes.

**Fig. 6.**
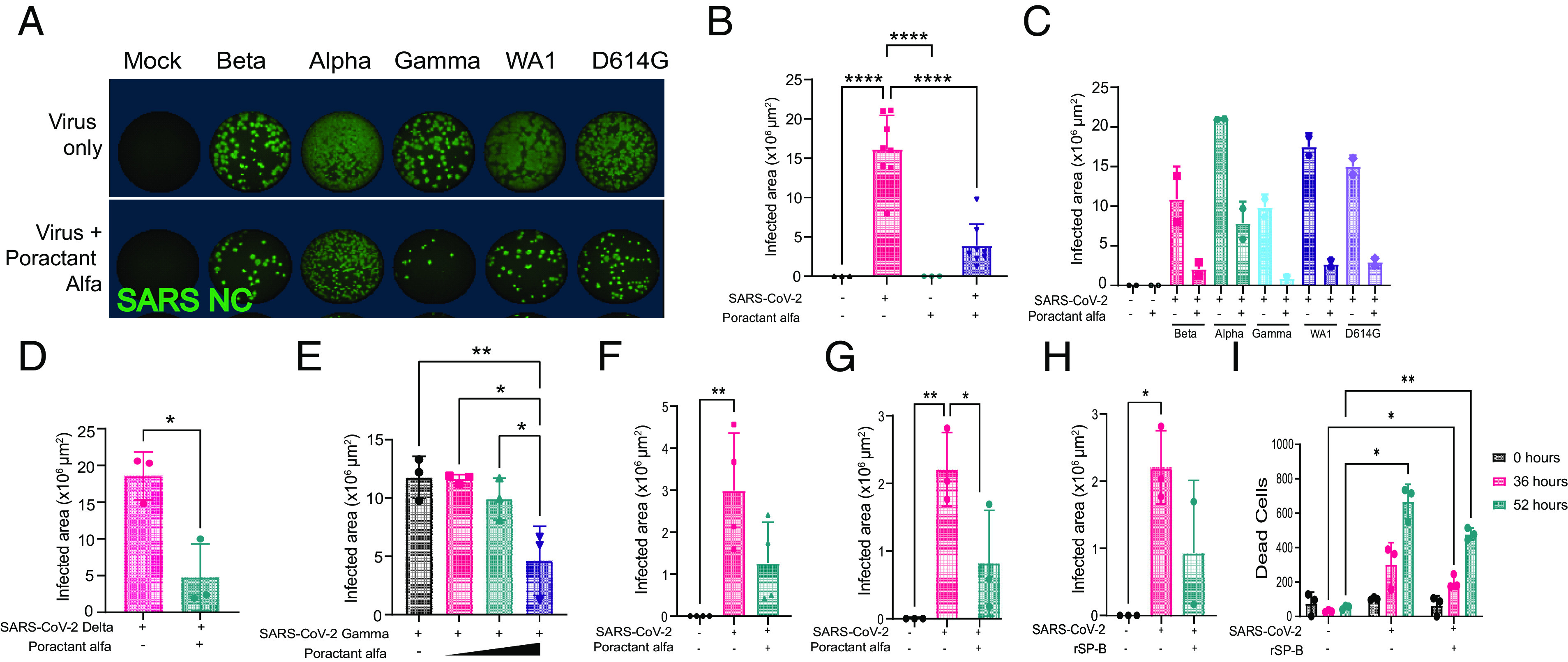
Natural surfactant and rSP-B reduce SARS-CoV-2 infection and cell death. (*A*) Immunofluorescence (via whole well scan) of Vero E6 cells 24 hpi with different variants of SARS-CoV-2 with or without the addition of poractant alfa. Wells were stained for intracellular viral NC to quantify degree of infection and quantified (FFU). (*B*) Quantification of data from (*A*) (all variants taken together). Surfactant dramatically diminishes infection. (*C* and *D*) Quantification of degree of infection of Vero E6 cells based on specific SARS-CoV-2 variant, with or without the addition of poractant alfa; (*C*) shows the results from variants Beta, Alpha, Gamma, WA1, and D614G; (*D*) shows the results from Delta. (*E*) Total infected Vero E6 cells 24 hpi after increasing dosages of poractant alfa; its antiviral actions increase with dose. (*F*–*I*) The addition of surfactant or rSP-B to SP-B-deficient PLOs and DLOs can rescue them from their otherwise higher susceptibility to infection and death. (*F*) Reiteration of the normal inhibition of infection of WT PLO cells with the addition of surfactant. The pivotal role of SP-B in this “rescue” is reinforced by showing that the otherwise higher rate of infection of SP-B-deficient DLO cells is diminished by the addition of surfactant (*G*) or rSP-B (*H*). Similarly, cell death (quantified using PI) of infected SP-B-deficient PLOs is reduced at 52 h by the addition of rSP-B (*I*). Significance calculated using two-way ANOVA: *P < 0.05, **P < 0.01, and ****P < 0.0001.

We next explored the clinically relevant implications of these observations. We tested whether an exogenous whole surfactant preparation, which is presently in clinical practice for newborns, could inhibit or reverse the pathogenic actions of SARS-CoV-2 (Fig. 6*A*). First, we performed dose-finding and timing studies by applying porcine-derived surfactant, poractant alfa (Curosurf) (80) (*SI Appendix,* Table S3) to Vero E6 cells 30 min prior to infection with the SARS-CoV-2 variants WA1, Alpha, Beta, Gamma, and D614 (Fig. 6*A*). Based on assessments of NC immunopositivity 24 hpi, there was a significant reduction in the number of SARS-CoV-2-infected cells in poractant alfa-treated cells compared to untreated (Fig. 6 *B*–*D*), with greater reductions in response to increasing surfactant concentrations (Fig. 6*E*). We then pretreated infected WT (Fig. 6*F*) and SP-B mutant DLOs (Fig. 6*G*) with poractant alfa and, similarly, found a significant reduction in the number of infected cells. To confirm that the underlying mechanism of therapeutic action of this practical clinical intervention pivoted on the expression of SP-B, we showed that adding rSP-B alone to SP-B-mutant DLOs similarly reduced infection and cell death at 36 and 54 hpi (Fig. 6 *H* and *I*).

## Discussion

The continued wide prevalence of COVID-19 and the persistence of symptoms in multiple organs (“long COVID”) remind us of how much remains unknown about the pathogenesis of, and host response to, SARS-CoV-2 and to viral infection broadly. Such understanding should enable better or adjunctive therapeutics to emerge. Here, we employed a unique human lung model system capable of determining, in a prospective and unbiased single-cell manner, the downstream transcriptional and functional responses of various pulmonary epithelial and mesenchymal cells upon initial exposure to multiple SARS-CoV-2 variants. Importantly, these assessments were conducted in a way that eliminated confounding factors from nonpulmonary organs or cell lineages, which could interfere if the lung were studied in situ. Hence, we could identify processes and responses that were lung-autonomous. Studying primary human lung cells in parallel to validate our findings, we were able to challenge prevailing views about COVID-19, as well as provide unique mechanistic insight into the lung’s response to viral incursion more generally. Importantly, such findings suggest at least two immediately actionable therapeutic strategies.

The initial striking finding was SARS-CoV-2's broader tropism for pulmonary cells than previously reported. Our scRNAseq data revealed viral entry and persistence that were *independent* of ACE2, TMPRSS2, and FURIN, but dependent on other entry routes mediated by cathepsin B/L/S and CD147/BSG. This transcriptomic picture was supported by immunocytochemical evidence of infected cells that lacked ACE2. While there have been reports of SARS-CoV-2 using receptors other than ACE2 to enter the cell (“receptor-mediated” or canonical), here we describe a noncanonical route: an *endocytotic* mechanism (*macropinocytosis*) employed by SARS-CoV-2 to enter a cell. The confirmed “back door” route, as shown in our apilimod assay (Fig. 2*M*), might potentially explain the infection of lung cells lacking ACE2 expression. This may also elucidate the persistent virulence of some SARS-CoV-2 variants despite effective abrogation of spike protein-mediated entry by vaccines or drugs. Our pharmacological identification and dissection of this unique macropinocytotic mechanism suggest an adjunctive therapy that might be used synergistically with others: apilimod, an FDA-approved drug that can be repurposed for clinical use to block this noncanonical entry route. While, in these experiments, we pretreated the cells with apilimod prior to infection (in part, as a tool for confirming the presence of macropinocytosis), suggesting its efficacy as a *prophylactic* agent, we thought that its ability to block noncanonical viral entry after infection may also constitute a *treatment* by limiting intercellular viral spread and viral load in COVID-19 patients, hence “short-circuiting” the disease. Although, theoretically, there might be concerns that apilimod’s mechanism of action could cause off-target blockade of some desirable immune responses or act deleteriously on PIKfyve elsewhere, no such manifestations have been observed following its use in >700 patients with non-Hodgkin’s lymphoma, autoimmune conditions, and even healthy volunteers, in whom the pharmacokinetic and safety profiles are FDA-acceptable.

While there is a rich literature describing lung inflammation following viral infection, such responses have involved the contribution of innate and adaptive immune cells. Infection of our lung epithelia, in the absence of hematopoietic derivatives or a blood supply, leads to immune-specific changes, activation of proinflammatory signaling, and the production of proinflammatory cytokines and chemokines. In other words, what we observed was an intrinsic, lung-autonomous, intrapulmonary, inflammatory response that provides the lung with baseline protective surveillance, but then, upon viral attack, is an acute “first responder” prior to the recruitment of hematopoietic derivatives. These data suggest that in response to SARS-CoV-2 infection, lung epithelial cells themselves can serve as initiators of innate immunity and impact gene expression related to oxidative stress tolerance, barrier cell maintenance, suppression of viral infection and dissemination, and support of cell survival. These epithelial-derived molecules attract both innate and adaptive immune cells from the systemic circulation, which, in some circumstances, might worsen the inflammatory milieu via a positive feedback loop. Armed with this greater mechanistic insight into the acute effects of SARS-CoV-2 on human lung cells, we might contemplate earlier ways of preempting or minimizing the fibrosis and smoldering inflammation that contributes to pulmonary long COVID. The key will be to optimize the efficacy of this intrapulmonary surveillance system for neutralizing an inciting virus, hence minimizing inflammatory feedback. Accomplishing such optimization requires knowing the key regulator of this inherent intrapulmonary host-pathogen defense mechanism, which highlights the third unexpected observation to emerge from using this LO system, described in the next paragraph.

This intrinsic, immediate intrapulmonary antiviral host defense system is orchestratedby SP-B, a molecule primarily known for its role in reducing alveolar surface tension, and now identified as having signaling and anti-inflammatory capabilities. Strikingly, we found that the expression of SP genes in LOs was dynamically altered by SARS-CoV-2 infection. Infectivity was greatest in SP-B-deficient LOs. Restoration of SP-B function and surfactant production in these LOs, via CRISPR-Cas9-mediated correction of the mutation, by prophylactic administration of rSP-B or exogenous surfactant, restored the LO’s ability to evince its inflammatory/innate immune/viral surveillance cascade, suppress viral entry and intercellular dissemination, and prevent viral-induced apoptosis. One could imagine that, in an actual patient, persistent viral infection could lead to surfactant depletion if consumption outstrips production, or if too many AT2 and club cells have died (which we had shown were targets for infection in our single-cell RNA seq data), enabling viral spread and inflammatory modes of cell death. Although our experiments were designed (as most antiviral drug studies are) to test the efficacy of surfactant to block acute infection, we believe that surfactant treatment could also be given *after* infection because it will dampen viral load and disease severity (reducing the risk of ARDS) while also helping to inhibit intercellular spread, hence truncating the viral life-cycle and preempting disease progression.

The actual mechanism by which surfactant reduces cellular infection is unclear. It may be due to its barrier function to viral entry, or to the creation of micelles that entrap viral particles. But our data suggest that its role is greater than these physical chemical actions, but rather significantly attributable to its modulatory effects on inflammatory mediators upstream of this intrapulmonary viral surveillance and defense system (73, 81–85). Indeed, in SP-B-deficient LOs, even baseline levels of immune surveillance cytokines are abnormally low.SP-B is controlled by multiple upstream regulators, including inflammatory mediators IL-1, IL-6, TGFβ and the transcription factors NKX2-1, JAK1 and STAT3 (*SI Appendix*, Fig. S10). Therefore, the absence of SP-B might be anticipated to exacerbate the deleterious components of the post-infection inflammatory response leading to increased pulmonary cell death and apoptosis.

The translational question is whether aggressive and timely surfactant repletion and/or SP-B replacement, might be therapeutic clinically following SARS-CoV-2 by inhibiting viral dissemination in the upper airway, modulating local immune responses and inflammatory cascades, preventing chronic inflammation, and reducing cell death, as we demonstrated in the LOs using surfactant-based therapeutics. Intratracheal administration of exogenous surfactant has been in clinical practice for decades in newborns, and exploratory trials are contemplated (86–88) or ongoing (NCT04375735) to test the efficacy of such preparations in mitigating COVID-19 ARDS. Having identified the critical role of surfactant in antiviral defense in the alveoli and airways, we now propose the use of aerosolized surfactants, which could prove lifesaving against acute COVID-19 (and other respiratory viruses) and potentially preempt aspects of long pulmonary COVID.

To elaborate on that last point, our observation that SPs play such an unanticipated but prominent role in the lung’s first response to viral infection may have implications beyond solely SARS-CoV-2. For example, we found that regardless of how the virus enters a cell—whether via the canonical receptor-mediated route or the noncanonical endocytotic route—the lung’s autonomous, intrinsic viral defense mechanism as mediated via SP signaling was evoked. It is plausible that enhancing these SP-associated actions downstream of viral entry may be an adjunctive strategy for bolstering the lung’s defense to a broad range of viral invaders, particularly in vulnerable patients.

COVID-19 hits deprived socioeconomic populations and certain racial groups with increased incidence and severity (89). Other than noting the greater prevalence in those groups of morbidities that predispose to COVID-19, most preclinical models and clinical trials to date have not offered much mechanistic insight into the causes of this disparity (90) nor how to approach it therapeutically. Our experimental platform is unique in that we can differentiate hiPSCs from men and women of various racial and ethnic backgrounds into LOs to determine differences in response to infection and/or therapeutic compounds in a patient-specific manner. While such a detailed exploration is beyond the scope of this study, our preliminary work-to-date using such diverse LOs has already offered observations that should be pursued using experimental systems like ours (*SI Appendix,* Fig. S5).

In summary, we have shown that acute infection of human LOs induces rapid changes in the innate immune responses via interferon signaling, oxidative stress, and hypoxia-inducible factor signaling. The influence of age, inflammation, and SP availability (79, 91) on canonical and noncanonical viral entry and intrapulmonary immunological/inflammatory cascades as unveiled in this study requires further investigation and suggests some potential interventions.

## Methods

LOs were generated from a diverse set of hiPSCs and infected with different variants of SARS-CoV-2 as intact 3D LOs or acutely dissociated monolayers. Infection was confirmed with transcriptional- and protein-based assays. Inflammation was determined via bulk and scRNA sequencing. Cell death was determined using BH3 mimetics. SP-B-deficient and CRISPR-corrected isogenic SP-B-deficient LOs were pretreated with exogenous surfactant and rSP-B before infection. Detailed Experimental Procedures are provided in *SI Appendix*.

## Supplementary Material

Appendix 01 (PDF)

## Data Availability

scRNA-seq data are available from the GEO repository database: GSE214762, GSE214752, and GSE214770 (92–94). RNA-seq data are available from the GEO repository database: GSE214482 (95). The custom-scripted macro used for automated image analysis of FFU imaging data is available at https://figshare.com/s/13587b74c09c251b3345 (96). (DOI for when we publish it live will be 10.26180/14073236 (97)). The following reagent was deposited by the Centers for Disease Control and Prevention and obtained through BEI Resources, NIAID, NIH: SARS-Related Coronavirus 2, Isolate USA-WA1/2020, NR-52281.
