# Supplementary material for: A therapy for suppressing canonical and noncanonical SARS-CoV-2 viral entry and an intrinsic intrapulmonary inflammatory response
Source: Proc Natl Acad Sci U S A. 2024 Jul 19;121(30):e2408109121. doi: 10.1073/pnas.2408109121 (PMC11287264; doi:10.1073/pnas.2408109121)
Supplement: Supplementary file 1 — Appendix 01 (PDF) [file pnas.2408109121.sapp.pdf]

SUPPLEMENTAL INFORMATION (SI) APPENDIX SECTION

I. SUPPLEMENTAL TABLES & FIGURES WITH LEGENDS
II. DETAILED EXPERIMENTAL PROCEDURES
III. DETAILED ACKNOWLEDGEMENTS
IV. REFERENCES

I. SUPPLEMENTAL TABLES & FIGURES WITH LEGENDS

Table S1: Induced pluripotent stem cell lines.

Cell Name	Ethnicity	Sex	Age	Tissue	Produced Method	Type	Passage	Source
HYRO103	Hispanic	Male	31	Hepatic fibroblast	Retroviral	iPSC	Not specified	ATCC
GM23450	Black/African American	Female	20	Skin	Retroviral	iPSC	29	Coriell
GM23720	White	Female	22	Peripheral vein	Episomal	iPSC	44	Coriell
Alda-31616	White	Male	25	Lymphoblastoid Cell line	Episomal	iPSC	10	Snyder Lab
hiPro133	White	Female	n/a	Skin	Retroviral	iPSC	12	CCRM
SP212-1-Cr3 Corr R11	White	Female	n/a	Skin	Retroviral	iPSC	20	CReM

iPSC= induced pluripotent stem cell

Table S2: SARS-CoV-2 viral reads from uninfected and infected proximal lung organoids.

Group	Viral Reads (%)
SP-B Mutant PLO uninfected	0
SP-B Mutant PLO uninfected	0
SP-B Mutant PLO uninfected	0
SP-B Mutant PLO + Delta variant 3 hpi	0.11
SP-B Mutant PLO + Delta variant 3 hpi	0.38
SP-B Mutant PLO + Delta variant 3 hpi	0.40
SP-B Mutant PLO + Delta variant 24 hpi	20.41
SP-B Mutant PLO + Delta variant 24 hpi	16.48
Wild type PLO uninfected	0
Wild type PLO uninfected	0
Wild type PLO uninfected	0
Wild type PLO + Delta variant 3 hpi	0.14
Wild type PLO + Delta variant 3 hpi	0.36
Wild type PLO + Delta variant 24 hpi	0.68
Wild type PLO + Delta variant 24 hpi	0.25
Wild type PLO + Delta variant 24 hpi	0.58

PLO = Proximal Lung organoid; hpi = hours post infection

Table S3. Profile of poractant alfa.

	**CUROSURF (portactant alfa)**
Source	Porcine
Phospholipid concentration (mg/mL)	76
Dipalmitoylphosphatidylcholine (mg/mL)	30
SP-B (mg/mL)	0.45
SP-C (μg/ protein/μmol L^{-1} phospholipid)	5.0-11.6
Additives	No
Manufacturing	
Organic solvent extraction	Yes
Liquid-gel chromatography	Yes

Adapted from https://curosurf.com/why-curosurf/surfactant-profiles/

Table S4: Materials used in lung differentiations.

Name of Material/ Equipment	Company	Catalog Number
**Cell Culture**		
12 well plates	Corning	3512
12-well inserts, 0.4um, translucent	VWR	10769-208
2-mercaptoethanol	Sigma-Aldrich	M3148
Accutase	Innovative Cell Tech	AT104
ascorbic acid	Sigma	A4544
B27 without retinoic acid	ThermoFisher	12587010
Bovine serum albumin (BSA) Fraction V, 7.5% solution	Gibco	15260-037
Dispase	StemCellTech	7913
DMEM/F12	Gibco	10565042
FBS	Gibco	10082139
Glutamax	Life Technologies	35050061
Ham's F12	Invitrogen	11765-054
HEPES	Gibco	15630-080
Iscove's Modified Dulbecco's Medium (IMDM) + Glutamax	Invitrogen	31980030
Knockout Serum Replacement (KSR)	Life Technologies	10828028
Matrigel	Corning	354230
Monothioglycerol	Sigma	M6145
mTeSR plus Kit (10/case)	Stem Cell Tech	5825
N2	ThermoFisher	17502048
NEAA	Life Technologies	11140050
Pen/strep	Lonza	17-602F
ReleSR	Stem Cell Tech	5872
RPMI1640 + Glutamax	Life Technologies	12633012
TrypLE	Gibco	12605-028
Y-27632 (Rock Inhibitor)	R&D Systems	1254/1
**Growth Factors/Small Molecules**		
Activin A	R&D Systems	338-AC
All-trans retinoic acid (RA)	Sigma-Aldrich	R2625
BMP4	R&D Systems	314-BP/CF
Br-cAMP	Sigma-Aldrich	B5386

CHIR99021	Abcam	ab120890
Dexamethasone	Sigma-Aldrich	D4902
Dorsomorphin	R&D Systems	3093
EGF	R&D Systems	236-EG
FGF10	R&D Systems	345-FG/CF
FGF7	R&D Systems	251-KG/CF
IBMX (3-Isobtyl-1-methylxanthine)	Sigma-Aldrich	I5879
SB431542	R&D Systems	1614
VEGF/PIGF	R&D Systems	297-VP/CF

Table S5: Recipes of Medias used in lung differentiations.

3D organoid induction medium (day 17-22)
Serum-free basal medium (see recipe) supplemented with:
FGF7 (10 ng/mL)
FGF10 (10 ng/mL)
CHIR99021 (3 μM)
EGF (10 ng/mL)

3D organoid branching medium (day 23-28)
3D organoid induction medium (see recipe) supplemented with:
All-trans retinoic acid (0.1 μM)
VEGF/PIGF (10 ng/mL)

3D organoid maturation medium (day 29-34)
3D organoid branching medium (see recipe) supplemented with:
Dexamethasone (50 nM)
Br-cAMP (100 μM)
IBMX (100 μM)

3D Proximal lung organoid medium (day17-30)
Serum-free basal medium (SFBM) supplemented with:
FGF10 (100ng/ml)
bFGF (250ng/ml)
Y27632 (10 μM)
Dexamethasone (50 nM)
Br-cAMP (100uM)
IBMX (100uM)

3D Distal lung organoid medium (day17-30)
Distal- Serum-free basal medium (SFBM) supplemented with:
CHIR99021 (3 μM)
SB431542 (10 μM)
FGF7 (10ng/ml)
RA (0.1uM)
Dexamethasone (50 nM)
Br-cAMP (100uM)
IBMX (100uM)

AFE induction medium (day 4-6)
Serum-free basal medium (see recipe) supplemented with:
SB431542 (10 μM)
Dorsomorphin (2 μM)

DE induction medium (day 1-3)
48.5 mL RPMI1640 + Glutamax
1 mL B27 without retinoic acid
500 μl HEPES (1%)
500 μl pen/strep
Human activin A (100 ng/mL)
CHIR99021 (5 μM) - only in the first 24 hours

LPC induction medium (day 7-16)
Serum-free basal medium (see recipe) supplemented with:
BMP4 (10 ng/mL)
All-trans retinoic acid (RA) (0.1 μM)
CHIR99021 (3 μM)

Quenching medium 50ml

49 mL DMEM/F12
1 mL FBS
500ul Pen/Strep

Serum-free basal medium (SFBM) 500ml
375 mL Iscove's Modified Dulbecco's Medium (IMDM) + Glutamax
125 mL Ham's F12
5 mL B27 without retinoic acid
2.5 mL N2
500 µl ascorbic acid, 50 mg/mL
19.5 µl monothioglycerol, 500 µg/mL
3.75 mL bovine serum albumin (BSA) Fraction V, 7.5% solution
5ml pen/strep

Distal Lung Organoid Serum-free base medium (DLO-SFBM) 500ml
475mL F12
16.65mL BSA
7.5mL HEPES
400µL $CaCl_2$
500µL ITS
5mL Pen/Strep
5ml B27 without retinoic acid

Stem cell passaging medium (day 0) 50ml
50 mL DMEM/F12 with Glutamax
13 mL Knockout serum replacement (KSR)
650 mL NEAA
130uL 2-mercaptoethanol
650uL pen/strep

Table S6. SARS-CoV-2 strains.

Strain Name	Pango Lineage	Greek Name	Original sample sequencing links
USA/WA1-2020	A	none "WA1"	GenBank: MN985325.1
USA/NY-PV08410/2020	B.1	none "D614G"	GenBank: MT370900.1 and GISAID: EPI_ISL_421374
hCoV-19/USA/CA_UCSD_5574/2020	B.1.1.7	Alpha	GISAID: EPI_ISL_751801
hCoV-19/South Africa/KRISP-K005325/2020	B.1.351	Beta	GISAID: EPI_ISL_678615 with changes during passage at BEI listed in BEI datasheet https://www.beiresources.org/Catalog/animalviruses/NR-54009.aspx
hCoV-19/Japan/TY7-503/2021	P.1	Gamma	GISAID: EPI_ISL_792683
hCoV-19/USA/PHC658/2021	B.1.617.2	Delta	none, changes from reference listed in BEI datasheet (https://www.beiresources.org/Catalog/animalviruses/NR-55611.aspx) and supplement to original publication (https://www.nejm.org/doi/suppl/10.1056/NEJMc2107799/suppl_file/nejmc2107799_appendix.pdf)
hCoV-19/USA/CA-SEARCH-59467/2021	BA.1.20	Omicron	GISAID: EPI_ISL_8186377

Table S7. Antibodies

Primary antibodies	Company	Catalogue #	Dilution rate
Nucleocapsid	GeneTex	GTX135357	1:1000
Spike antibody	GeneTex	GTX632604	1:1000
ACE2	Cell signaling	4355S	1:500
MUC5AC	Millipore	MAB2011	1:200
SOX9	R&D Systems	AF3075	1:200
SCGB3A2	abcam	ab181853	1:300
HOPX	Santa Cruz Biotech	sc-398703	1:200
proSPC	abcam	ab40871	1:250
AcTub	Cell Signaling	5335	1:600
SPC (mature)	LS Bio	LS-B9161	1:300
SPB (mature)	7 Hills	48604	1: 500 (I) 1:500 (W)[a]
SPA	Abcam	Ab51891	1:400
SPD	Abcam	Ab203309	1:400
HTI-56	Terrace Biotech	TB-29AHT1-56	1:150
p63	Boster	PB9152	1:250
E-cadherin	R&D Systems	AF748	1:100
SMA	Invitrogen	50976080	1:100

Secondary Antibodies	Company	Catalogue #	Dilution rate
Donkey anti-mouse IgG (Alexa 488)	Abcam	Ab150105	1:500
Donkey anti-mouse IgG (Alexa 555)	Abcam	Ab150106	1:500
Donkey anti-mouse IgG (Alexa 647)	Abcam	Ab150107	1:500
Donkey anti-rabbit IgG (Alexa 488)	Abcam	Ab150073	1:500
Donkey anti-rabbit IgG (Alexa 555)	Abcam	Ab150074	1:500
Donkey anti-rabbit IgG (Alexa 647)	Abcam	Ab150075	1:500
Donkey anti-goat IgG (Alexa 488)	Abcam	Ab150129	1:500
Donkey anti-goat IgG (Alexa 555)	Abcam	Ab150130	1:500
Donkey anti-goat IgG (Alexa 647)	Abcam	Ab150131	1:500

FIGURE S1: Differentiation of human pluripotent stem cells (hPSC) into multi-cell type 3D lung organoids (LOs). (A) Schematic for directed differentiation of hPSCs into 3D LOs with corresponding phase contrast images of each step, including undifferentiated hPSCs, definitive endoderm, anterior foregut endoderm, and lung progenitor cells (LPC). Image of 3D LPCs shows NKX2-1 via GFP expression. (B) Live cell phase contrast images of the representative morphology of 3 different 3D lung organoid types: proximal (PLO), distal (DLO) and whole lung organoids (WLO). (C) Immunostaining of dissociated 3D WLOs with some representative lung epithelial and mesenchymal markers. Their respective cell types are shown in the schematic in (D). (D) Schematic of the epithelial cells in the PLOs and DLOs with representative immunofluorescent images of the epithelial markers found in the DLOs (alveolar type 2 cells) and PLOs (airway cells).

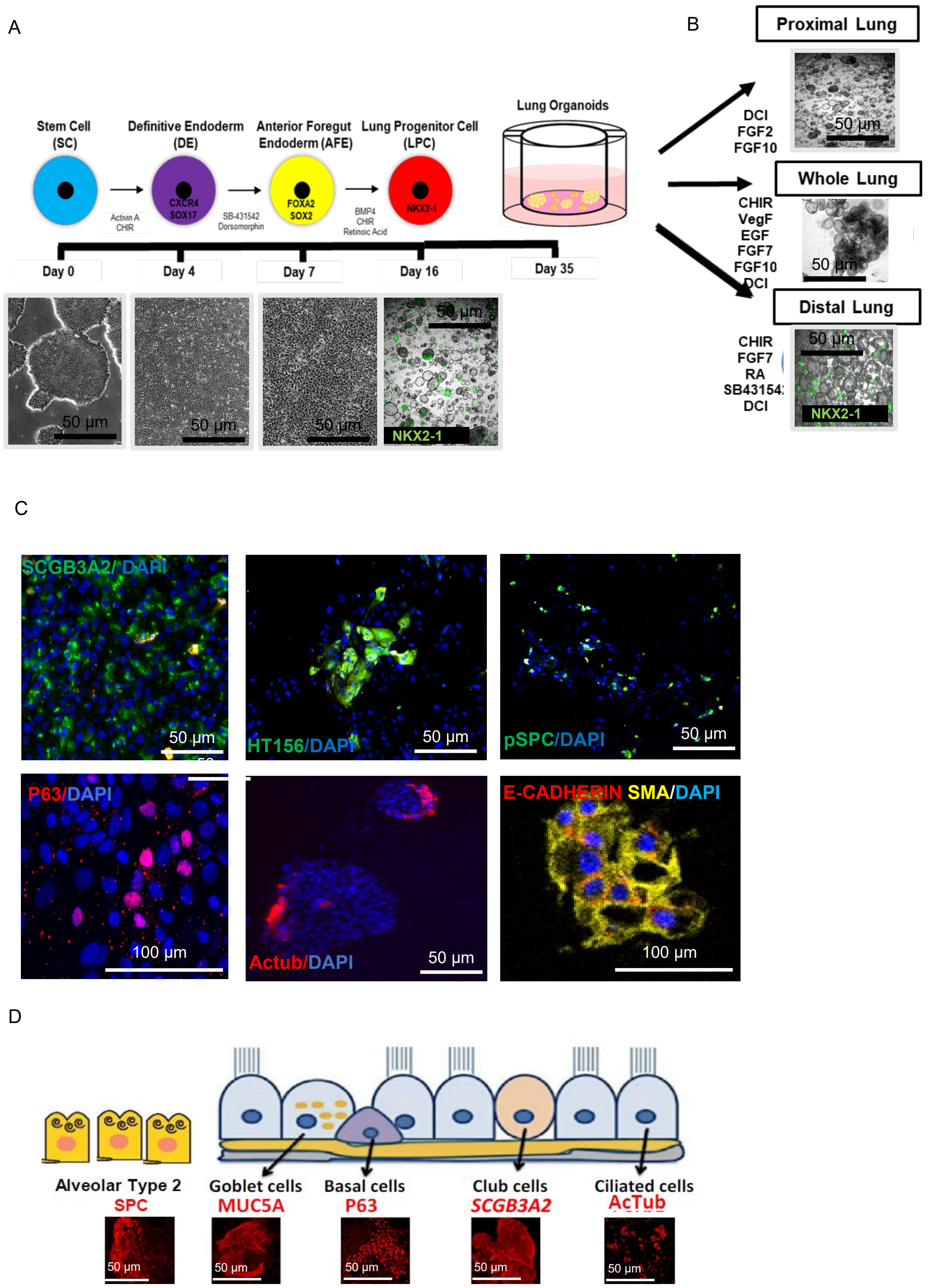
A
Stem Cell (SC)
Definitive Endoderm (DE)
Anterior Foregut Endoderm (AFE)
Lung Progenitor Cell (LPC)
Lung Organoids
CXCR4 SOX17
FOXA2 SOX2
NKX2-1
Activin A CHIR
SB-431542 Dorsomorphin
BMP4 CHIR Retinoic Acid
Day 0
Day 4
Day 7
Day 16
Day 35
50 µm
NKX2-1
B
Proximal Lung
DCI FGF2 FGF10
Whole Lung
CHIR VegF EGF FGF7 FGF10 DCI
Distal Lung
CHIR FGF7 RA SB431542 DCI
C
SCGB3A2/ DAPI
HT156/DAPI
pSPC/DAPI
P63/DAPI
Actub/DAPI
E-CADHERIN SMA/DAPI
100 µm
D
Alveolar Type 2
SPC
Goblet cells
MUC5A
Basal cells
P63
Club cells
SCGB3A2
Ciliated cells
AcTub

FIGURE S1

FIGURE S2: Transcriptional characterization of LOs. (A) Uniform manifold approximation and projection (UMAP) of hPSC-DLOs and characteristic genes per cluster. (B) UMAP of hPSC-WLOs and characteristic genes per cluster.

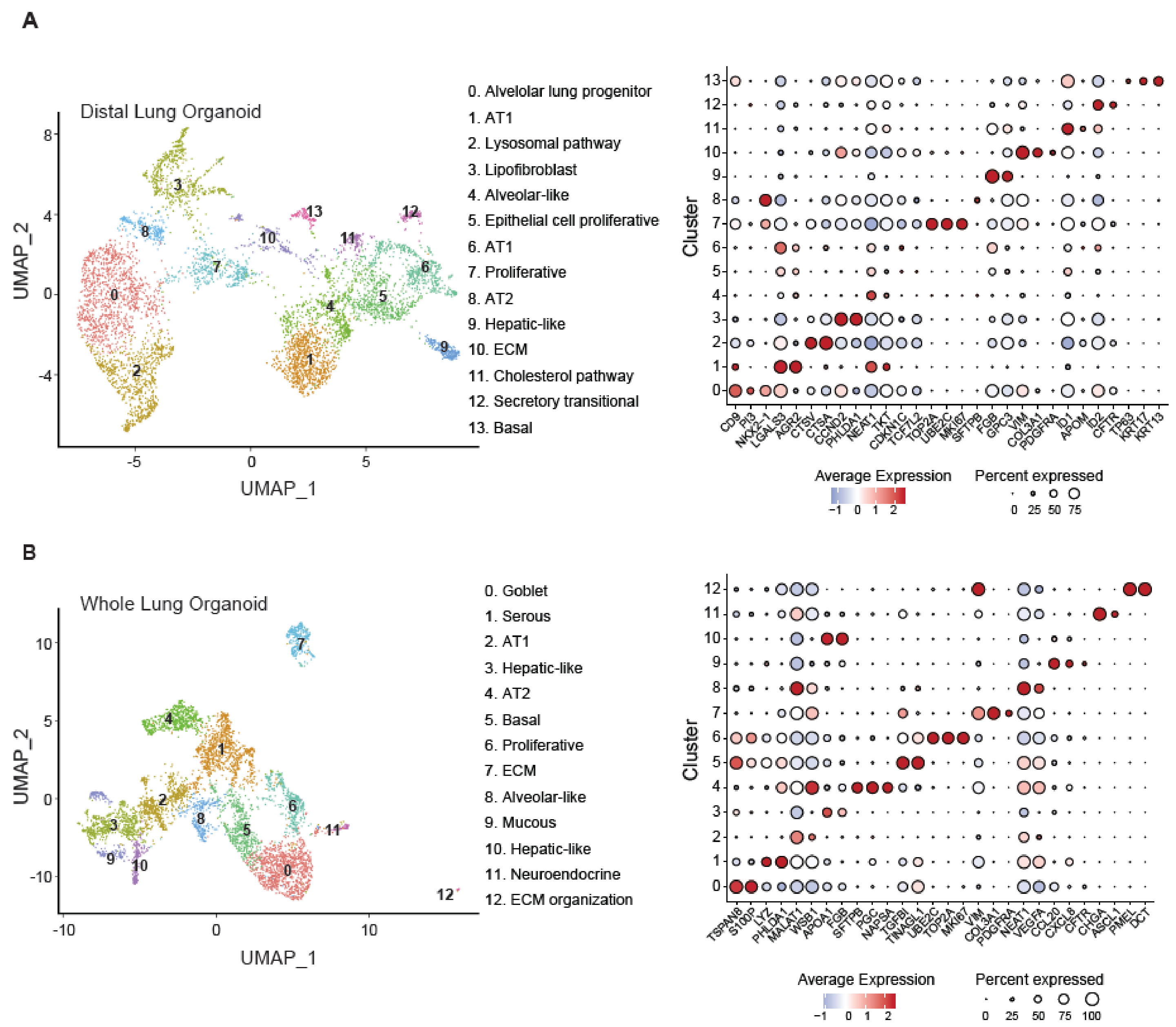
A
Distal Lung Organoid
UMAP_1
UMAP_2
0. Alvelolar lung progenitor
1. AT1
2. Lysosomal pathway
3. Lipofibroblast
4. Alveolar-like
5. Epithelial cell proliferative
6. AT1
7. Proliferative
8. AT2
9. Hepatic-like
10. ECM
11. Cholesterol pathway
12. Secretory transitional
13. Basal
Cluster
CD9 PI3 NKX2-1 LGALS3 AGR2 CTSV CTSA CCND2 PHLDA1 NEAT1 TKT CDKN1C TCF7L2 TOP2A UBE2C MKI67 SFTPB FGB GPC3 VIM COL3A1 PDGFRA ID1 APOM ID2 CFTR TP63 KRT17 KRT13
Average Expression
Percent expressed
B
Whole Lung Organoid
UMAP_1
UMAP_2
0. Goblet
1. Serous
2. AT1
3. Hepatic-like
4. AT2
5. Basal
6. Proliferative
7. ECM
8. Alveolar-like
9. Mucous
10. Hepatic-like
11. Neuroendocrine
12. ECM organization
Cluster
TSPAN8 S100P LYZ PHLDA1 MALAT1 WSB1 APOA1 FGB SFTPB PGC NAPSA TGFBI TINAGL1 UBE2C TOP2A MKI67 VIM COL3A1 PDGFRA NEAT1 VEGFA CCL20 CXCL8 CFTR CHGA ASCL1 PMEL DCT
Average Expression
Percent expressed

FIGURE S2

FIGURE S3: Infection of hPSC-derived lung organoids (LOs) with SARS-CoV-2 pseudovirus. (A) Schematic for SARS-CoV-2 pseudovirus infection in human lung organoids (LO). LOs were cultured in serum free basal medium and infected with SARS-CoV-2 pseudovirus for 24 hours. (B) Representative immunostaining of live, intact 3D PLOs with goblet cell marker MUC5AC and pseudovirus-GFP. (C) Dual immunostaining of 3D PLOs (acutely dissociated to enable visualization of individual cells in monolayer without extensive overlapping of cells) for both identifying lung epithelial markers and pseudovirus-GFP.

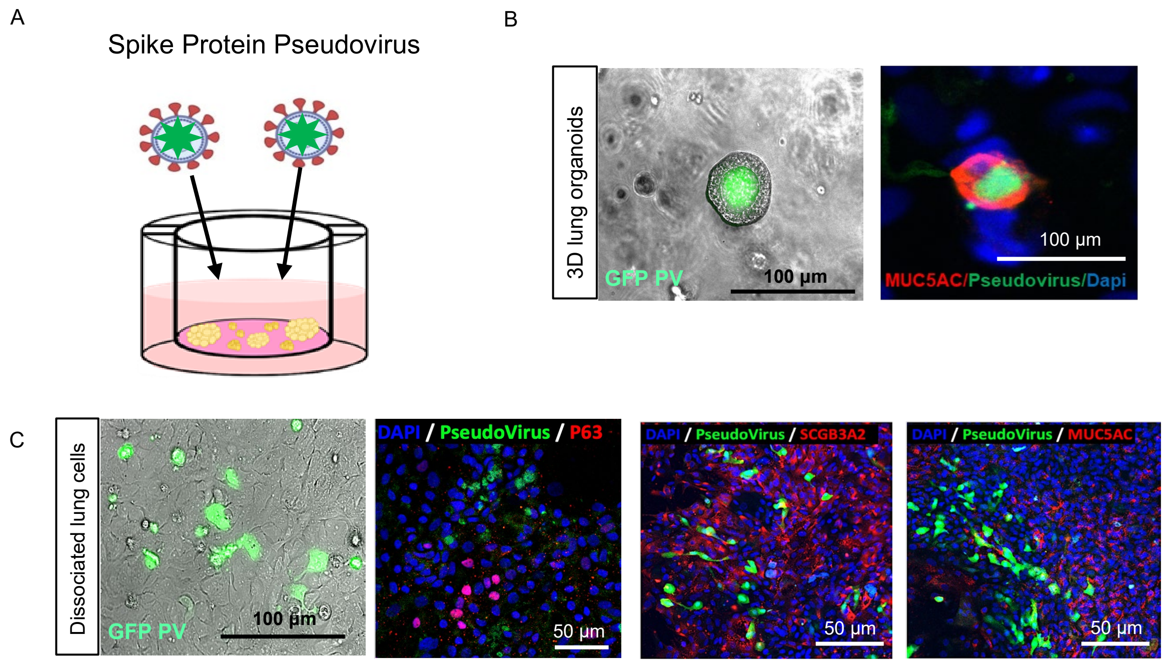
A
Spike Protein Pseudovirus
B
3D lung organoids
GFP PV
100 µm
100 µm
MUC5AC/Pseudovirus/Dapi
C
Dissociated lung cells
GFP PV
100 µm
DAPI / PseudoVirus / P63
50 µm
DAPI / PseudoVirus / SCGB3A2
50 µm
DAPI / PseudoVirus / MUC5AC
50 µm

FIGURE S3

FIGURE S4: Infection of human lung organoids with SARS-CoV-2. (A) FFU titers of PLO (Prox) and DLO (Dist) supernatant. (B) Ingenuity pathway analysis (IPA) canonical pathways comparing the gene expression patterns of WT vs. SP-B deficient PLOs 24 hours post infection (hpi) with a replication-competent SARS-CoV-2 virus vs. mock-infected controls (C) SARS-CoV-2 viral genes in each cluster as violin plots. The vertical bars represent the expression level. Note that every cluster expresses viral genes. (D) IFNome90 gene set enrichment scores of 24 hpi vs mock infected wild type and SP-B deficient PLOs. Positive NES defines interferon enrichment.

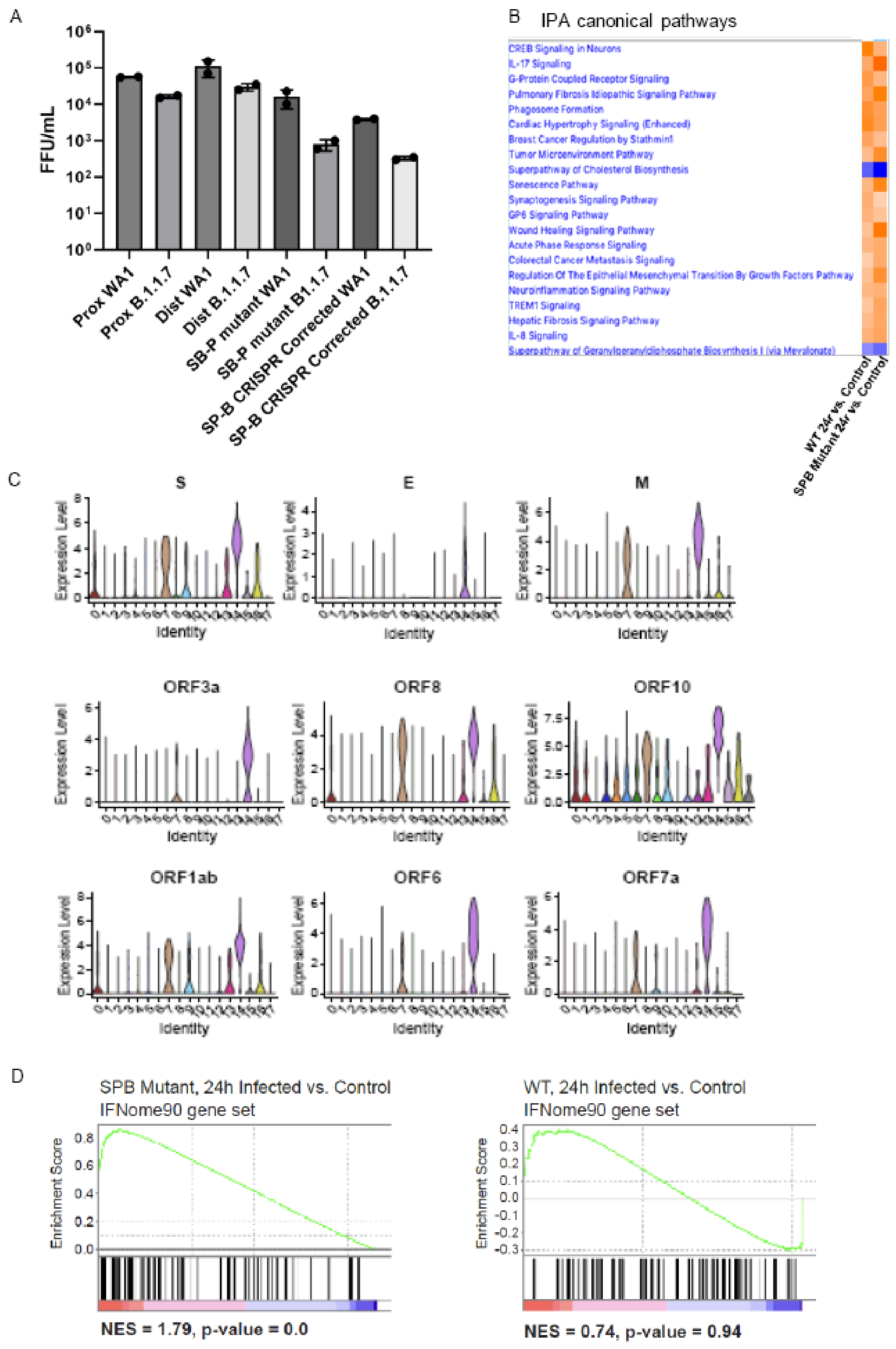
A
FFU/mL
Prox WA1
Prox B.1.1.7
Dist WA1
Dist B.1.1.7
SB-P mutant WA1
SB-P mutant B1.1.7
SP-B CRISPR Corrected WA1
SP-B CRISPR Corrected B.1.1.7
B
IPA canonical pathways
CREB Signaling in Neurons
IL-17 Signaling
G-Protein Coupled Receptor Signaling
Pulmonary Fibrosis Idiopathic Signaling Pathway
Phagosome Formation
Cardiac Hypertrophy Signaling (Enhanced)
Breast Cancer Regulation by Stathmin1
Tumor Microenvironment Pathway
Superpathway of Cholesterol Biosynthesis
Senescence Pathway
Synaptogenesis Signaling Pathway
GP6 Signaling Pathway
Wound Healing Signaling Pathway
Acute Phase Response Signaling
Colorectal Cancer Metastasis Signaling
Regulation Of The Epithelial Mesenchymal Transition By Growth Factors Pathway
Neuroinflammation Signaling Pathway
TREM1 Signaling
Hepatic Fibrosis Signaling Pathway
IL-8 Signaling
Superpathway of Geranylgeranyldiphosphate Biosynthesis I (via Mevalonate)
WT 24r vs. Control
SPB Mutant 24r vs. Control
C
S
E
M
ORF3a
ORF8
ORF10
ORF1ab
ORF6
ORF7a
Expression Level
Identity
D
SPB Mutant, 24h Infected vs. Control
IFNome90 gene set
Enrichment Score
NES = 1.79, p-value = 0.0
WT, 24h Infected vs. Control
IFNome90 gene set
NES = 0.74, p-value = 0.94

FIGURE S4

FIGURE S5: Compounds blocking various routes of viral entry and processing show different efficacies in reducing viral infections. Western blot of PLOs from different racial groups, pre-treated with the endocytic blocking agent (apilimod [“API”]), the cathepsin B blocking agent (ONO5334 [“ONO”]), or an agent that blocks viral replication (Remdesivir [“REM”]) 24 hpi with SARS-CoV-2 (“V”). The data are representative of three independent experiments. PLO GM23450 was derived from hiPSCs generated from an African-American female, HYRO103 from a Hispanic male, GM2370 from a Caucasian female, and ALDA31616 from a Caucasian male (**Supplementary Table 1**). Actin served as a loading control. Interestingly, even at this cursory level of analysis, one can see the suggestion of possible disparity in the greater degree of infection (the DMSO control lane) between the Hispanic male and the others, as well as a disparate response to anti-viral agents: Apilimod may have worked less effectively in the Hispanic male and Remdesivir may have worked most effectively in the Caucasian male.

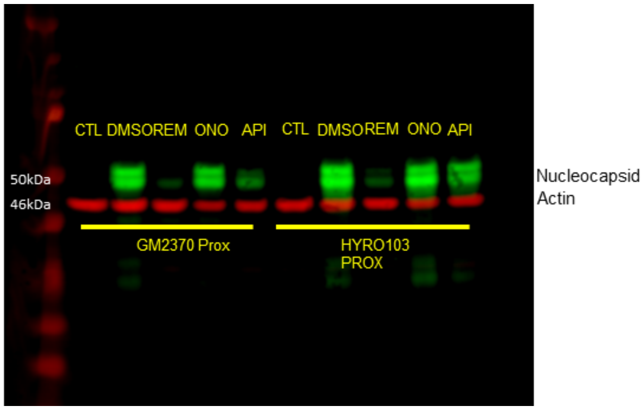
CTL DMSO REM ONO API CTL DMSO REM ONO API
50kDa
46kDa
Nucleocapsid
Actin
GM2370 Prox
HYRO103
PROX

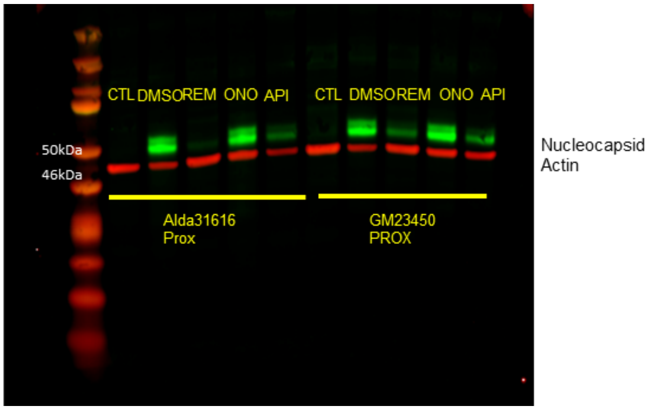
CTL DMSO REM ONO API CTL DMSO REM ONO API
50kDa
46kDa
Nucleocapsid
Actin
Alda31616
Prox
GM23450
PROX

FIGURE S5

FIGURE S6: Single cell transcriptional characteristics of the hPSC-LOs. (A) Uniform manifold approximation and projection (UMAP) of hPSC-PLOs 16 hpi and (B) characteristic genes per cluster.

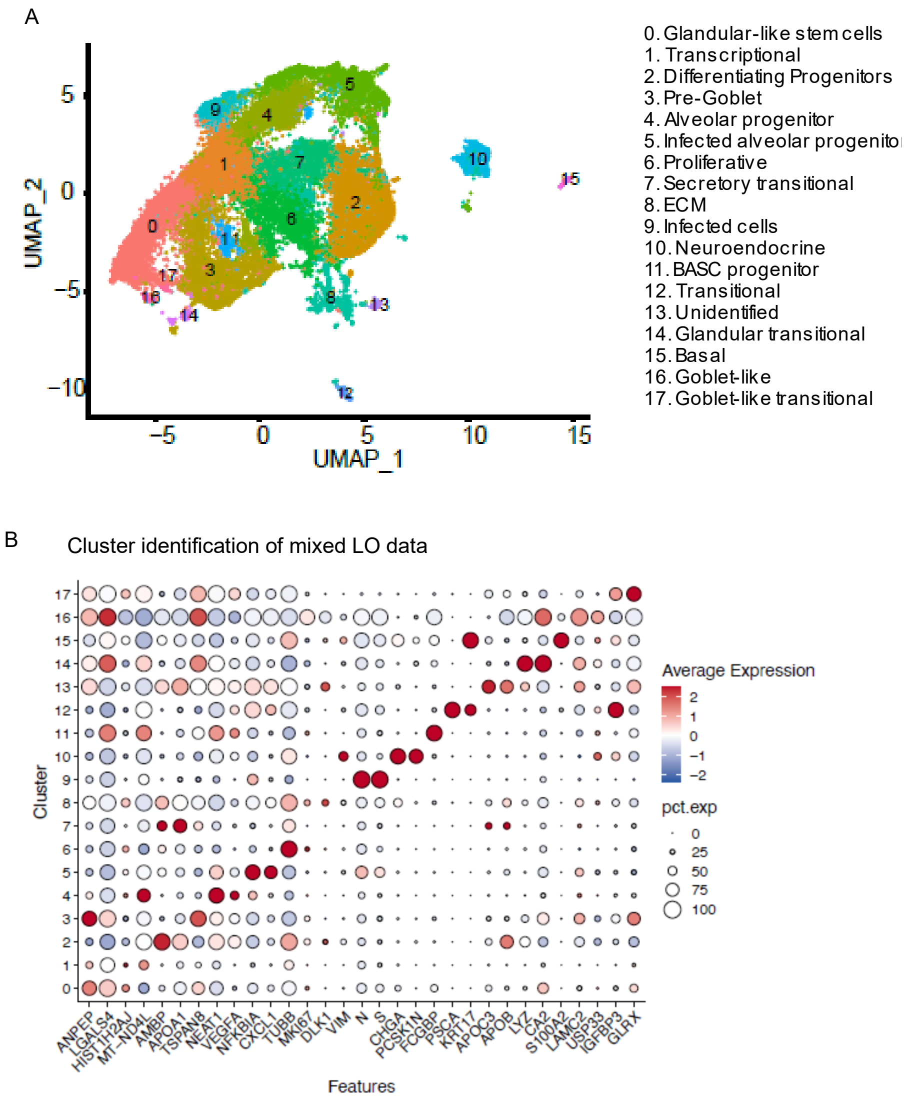
A
0. Glandular-like stem cells
1. Transcriptional
2. Differentiating Progenitors
3. Pre-Goblet
4. Alveolar progenitor
5. Infected alveolar progenito
6. Proliferative
7. Secretory transitional
8. ECM
9. Infected cells
10. Neuroendocrine
11. BASC progenitor
12. Transitional
13. Unidentified
14. Glandular transitional
15. Basal
16. Goblet-like
17. Goblet-like transitional
UMAP_1
UMAP_2
B
Cluster identification of mixed LO data
Average Expression
pct.exp
Cluster
Features
ANPEP
LGALS4
HIST1H2AJ
MT-ND4L
AMBP
APOA1
TSPAN8
NEAT1
VEGFA
NFKBIA
CXCL1
TUBB
MKI67
DLK1
VIM
N
S
CHGA
PCSK1N
FCGBP
PSCA
KRT17
APOC3
APOB
LYZ
CA2
S100A2
LAMC2
USP33
IGFBP3
GLRX

FIGURE S6

FIGURE S7: Acute infection with SARS-CoV-2 induces a lung-autonomous intrapulmonary inflammatory cascades. (A, B) UMAPs of a subset of interferon genes differentially-expressed in (A) mock vs (B) infected LOs. Highest expression levels are in red. (C) Dot plot showing differentially-expressed chemokine and cytokine genes by cluster, comparing infected vs mock LOs. See text for significance of each. (D) Canonical pathway analysis of infected vs mock LOs by cluster using IPA. (E) Disease and Biological Functions using IPA. (F) Upstream Regulators using IPA.

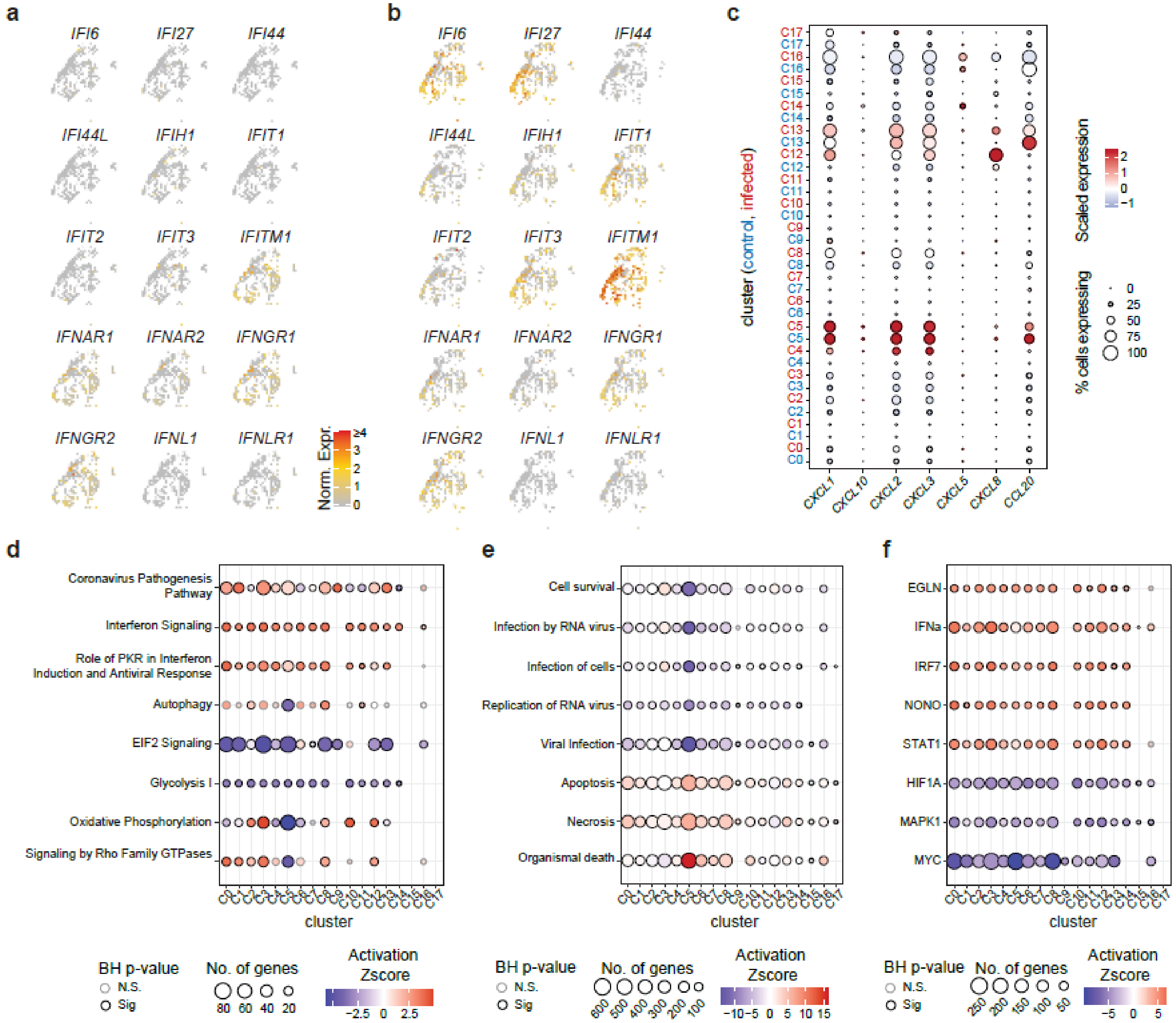
a
IFI6
IFI27
IFI44
IFI44L
IFIH1
IFIT1
IFIT2
IFIT3
IFITM1
IFNAR1
IFNAR2
IFNGR1
IFNGR2
IFNL1
IFNLR1
Norm. Expr.
b
c
cluster (control, infected)
CXCL1
CXCL10
CXCL2
CXCL3
CXCL5
CXCL8
CCL20
Scaled expression
% cells expressing
d
Coronavirus Pathogenesis Pathway
Interferon Signaling
Role of PKR in Interferon Induction and Antiviral Response
Autophagy
EIF2 Signaling
Glycolysis I
Oxidative Phosphorylation
Signaling by Rho Family GTPases
cluster
BH p-value
N.S.
Sig
No. of genes
Activation Zscore
e
Cell survival
Infection by RNA virus
Infection of cells
Replication of RNA virus
Viral Infection
Apoptosis
Necrosis
Organismal death
f
EGLN
IFNa
IRF7
NONO
STAT1
HIF1A
MAPK1
MYC

FIGURE S7

FIGURE S8: Transcriptional changes and tropism in SP-B deficient hiPSC-LOs 16 hpi with SARS-CoV-2 (A) UMAP of 3D SP-B deficient PLOs, 24 hpi with the Alpha variant. Ratios of each cluster in the wild type and SP-B mutant PLOs are presented in the right-hand figure. Clusters were generated via Harmony Integration of 1 SP-B deficient PLO and 3 wild type PLO independent samples. ECM = extracellular matrix (B) SARS-CoV-2 viral genes in each cluster from the SP-B deficient PLOs as violin plots. The vertical bars represent the expression level. (C) Violin plots of SARS-CoV-2 entry genes in each cluster in the SP-B deficient PLOs. Most cells in the LOs get infected by SARS-CoV-2 with viral entry either via a canonical route (ACE2 and TMPRSS2) or a non-canonical route (e.g., endocytosis/macropinocytosis).

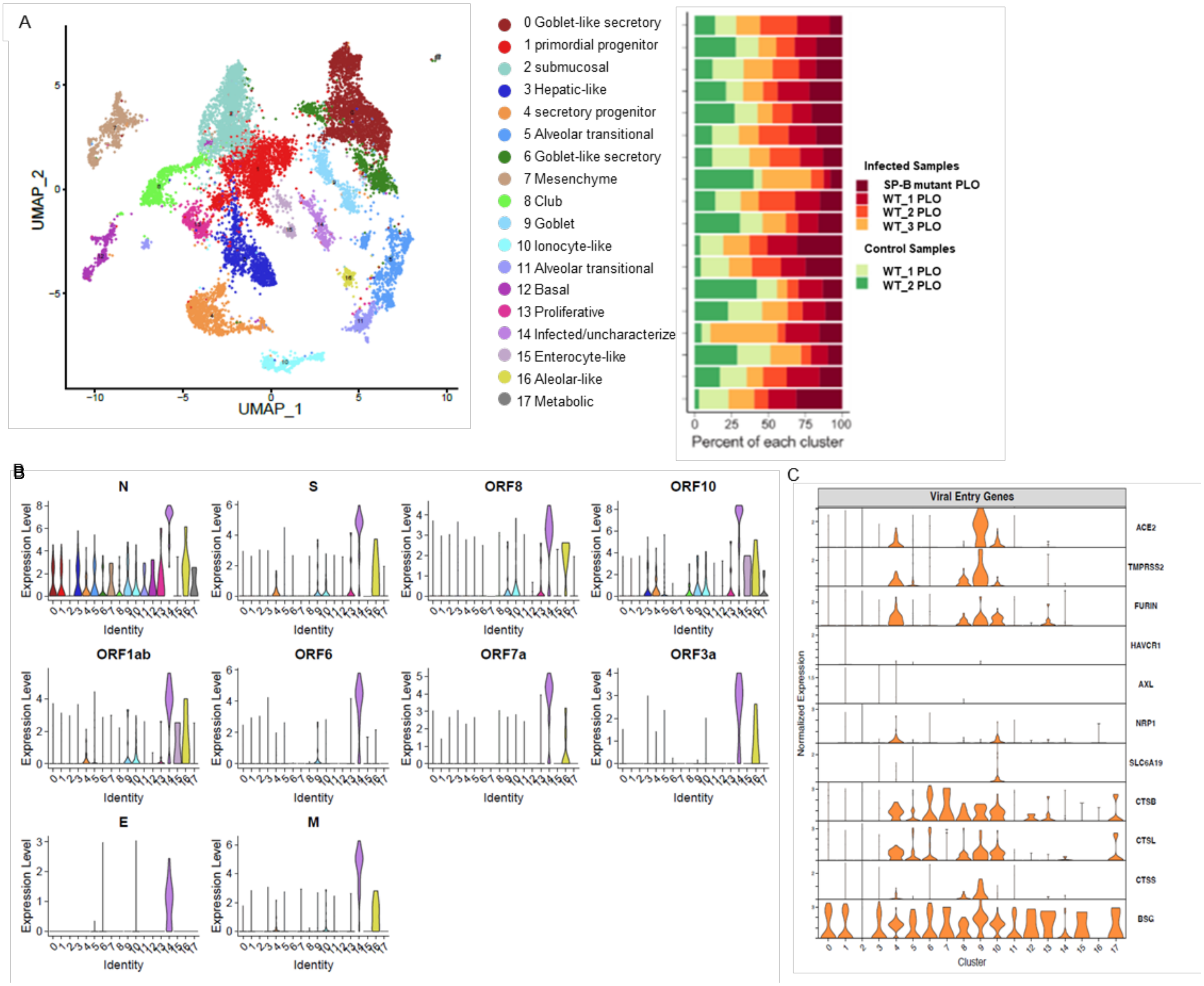
A
UMAP_1
UMAP_2
0 Goblet-like secretory
1 primordial progenitor
2 submucosal
3 Hepatic-like
4 secretory progenitor
5 Alveolar transitional
6 Goblet-like secretory
7 Mesenchyme
8 Club
9 Goblet
10 Ionocyte-like
11 Alveolar transitional
12 Basal
13 Proliferative
14 Infected/uncharacterize
15 Enterocyte-like
16 Aleolar-like
17 Metabolic
Infected Samples
SP-B mutant PLO
WT_1 PLO
WT_2 PLO
WT_3 PLO
Control Samples
WT_1 PLO
WT_2 PLO
Percent of each cluster
B
N
S
ORF8
ORF10
ORF1ab
ORF6
ORF7a
ORF3a
E
M
Expression Level
Identity
C
Viral Entry Genes
ACE2
TMPRSS2
FURIN
HAVCR1
AXL
NRP1
SLC6A19
CTSB
CTSL
CTSS
BSG
Normalized Expression
Cluster

FIGURE S8

FIGURE S9: Modulation of viability and viral dissemination following SARS-CoV-2 infection by BH3 mimetics. (a) Live cell imaging of 3D lung organoids by phase contrast imaging showing morphological changes triggered by SARS-CoV-2 (MOI 1) infection or mock infection controls. Images collected every 24h by Incucyte S3. (B) FFU immunoassay of SARS-CoV-2 infected VeroE6 cells (MOI:0.01) exposed to ABT-737 (250nM-2uM), A-1331852 (250nM-2uM), or Q-VD-OPh (10uM), and overlayed with 1.2% carboxymethylcellulose in media. Immunostaining of SARS-CoV-2 nucleocapsid in 96 well plates and counterstained with Hoechst. Representative of 3 independent experiments.

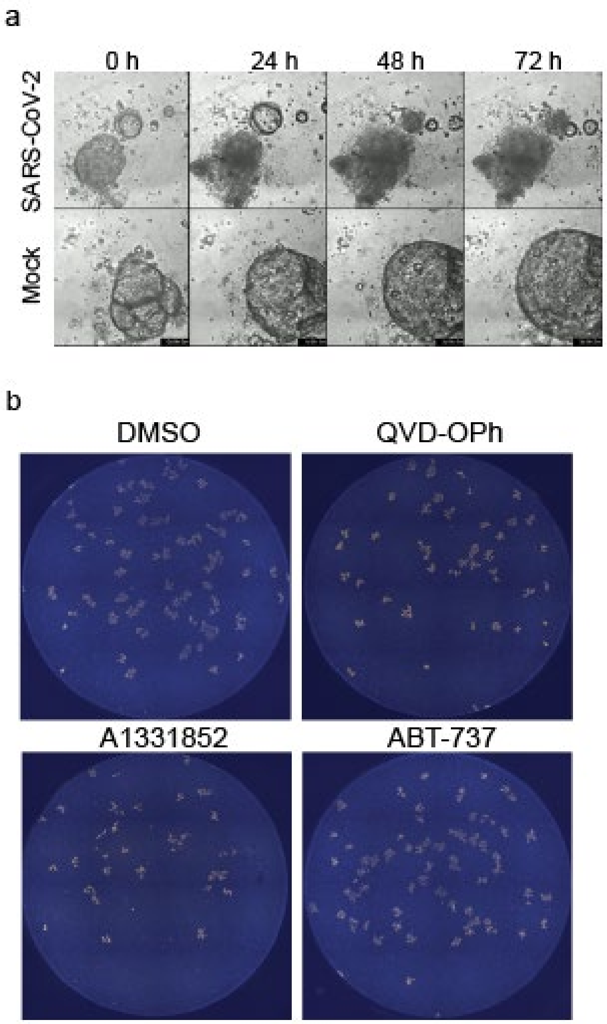

FIGURE S9

FIGURE S10: Transcriptional changes in wild type (WT) and SP-B deficient PLOs 24 hpi. Heat map showing average expression (represented as z-score) of key regulatory and transcriptional genes known to interact with surfactant protein B in WT and SP-B deficient 3D PLOs at 24 hpi with SARS-CoV-2. The genes differentially expressed between the infected wild type and SP-B deficient 3D PLOs are shown in green.

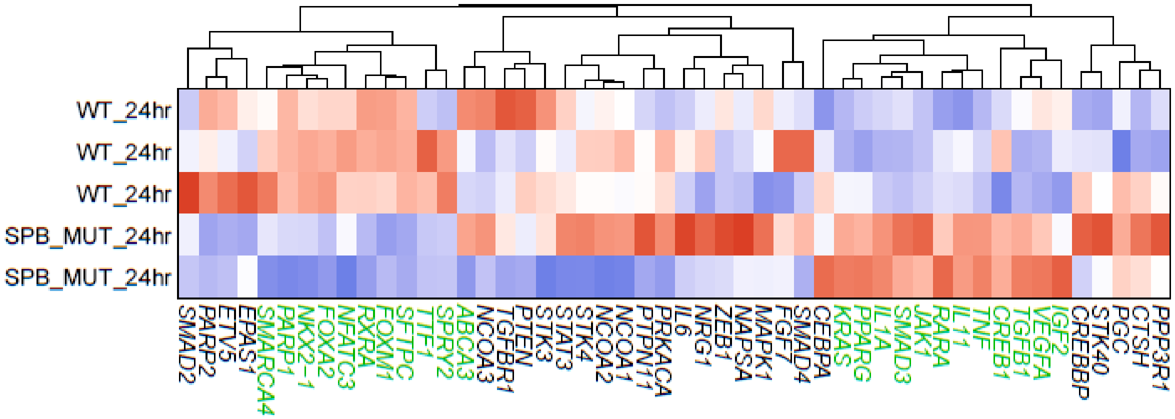
WT_24hr
WT_24hr
WT_24hr
SPB_MUT_24hr
SPB_MUT_24hr
SMAD2
PARP2
ETV5
EPAS1
SMARCA4
PARP1
NKX2-1
FOXA2
NFATC3
RXRA
FOXM1
SFTPC
TTF1
SPRY2
ABCA3
NCOA3
TGFBR1
PTEN
STK3
STAT3
STK4
NCOA2
NCOA1
PTPN11
PRKACA
IL6
NRG1
ZEB1
NAPSA
MAPK1
FGF7
SMAD4
CEBPA
KRAS
PPARG
IL1A
SMAD3
JAK1
RARA
IL11
TNF
CREB1
TGFB1
VEGFA
IGF2
CREBBP
STK40
PGC
CTSH
PPP3R1

FIGURE S10

II. DETAILED EXPERIMENTAL PROCEDURES

Cell Lines

Vero E6, A549, SK-LU-1, Caco2, and Calu-3 cell lines were obtained from ATCC. Huh7.5 cells were obtained from Apath LLC. TMPRSS2-VeroE6 were obtained from Sekisui XenoTech. A549, SK-LU-1, Caco2, and Calu-3 cells were propagated in MEM (Corning), 10% FBS, Penicillin-Streptomycin (Gibco). Vero E6, TMPRSS2-VeroE6, and Huh7.5 cells were propagated in DMEM (Corning) with 10% FBS and Penicillin-Streptomycin (Gibco) with Geneticin added for TMPRSS2-VeroE6. A549 cells were transduced with hACE2 lentivirus (ASMBio, Lot#200723) or firefly luciferase lentivirus (AMSBio, Lot #200325) with an MOI of 8 with 8mg/mL of polybrene. Transduced cells were selected with 1mg/mL puromycin for 3 days.

Human Embryonic Stem cell and human induced pluripotent stem cell lines were used in accordance with guidelines provided by the National Institutes of Health (NIH). **Supplementary table 1** highlights the human iPSCs used and how they were derived. The SP-B mutant lines were previously published (7). These cell lines have been validated by immunofluorescence for markers of pluripotency, karyotype, and genomic analysis if applicable. The hiPSCs and the human embryonic stem cell line H9 (WiCell), were cultured on matrigel coated (Matrigel GFR; Corning, #354230) plates in mTeSR medium (StemCell Technologies #85850). Medium was changed daily, and cells were passed using ReLeSR (StemCell Technologies #05872) every 5–7 days. Cultures were maintained in an undifferentiated state in a 5% CO2/ambient air environment.

Authentication of Cell Lines is based on guidelines from the International Cell Line Authentication Committee (iclac.org): Authentication testing was performed on established cell lines regardless of the application. The pluripotency of the ESC and iPSC cell lines were confirmed through immunofluorescence for pluripotent markers (Oct4, Nanog, TRA1-81, SOX2, TRA 1-60) and normal karyotype was also confirmed prior to use. Early passages at the time of verification of pluripotency and karyotype are cryopreserved and those stocks utilized for further experimentation.

Different cell lines and derivatives were never manipulated together to avoid the possibility of cross-contamination. Furthermore, all cell lines were used at early passage. Upon reaching higher passage number, cells were discarded, and cultures restarted from early passage cryovials. Monthly screening for mycoplasma using the MyoSEQ ThermoFisher Mycoplasma Detection kit was performed, and new cells/cell lines were maintained in quarantine until confirmed to be mycoplasma negative.

Primary normal human bronchial epithelial cells (NHBECs) were sourced from Lonza (NHBE CC-2540; Walkersville, MD). These Lonza NHBECs were obtained from a 65-year-old Caucasian male without identifiers. The NHBECs provided were tested and certified negative for HIV, HBV, HCV, sterility, and mycoplasma. The NHBECs were expanded with

PneumaCult™ Ex-Plus media (StemCell #05040, Tukwila, WA). Cells were seeded at $5x10^6$ cells/ T75 flask and incubated at 37°C, 5% CO_2. Culture media was changed every other day. Once cells reached 70-80% confluency, they were dissociated using Animal Component-Free Cell Dissociation kit (StemCell #05426, Tukwila, WA). When cells were ~70-80% confluent, they were dissociated and seeded on collagen-coated 6.5 mm transwells (Corning #29442-082, VWR). Transwells were pre-coated with 50µg/mL Collagen type I from Rat tail (BD Biosciences #354236) at 7.5 µg/cm^2. Collagen-coated transwells were rehydrated with 100 µL PneumaCult™ Ex-Plus media at 37°C, 5% CO_2 for 30 min prior to seeding NHBECs for ALI culture. NHBECs were then seeded in the apical chamber at $5x10^4$ cells in 100uL of media, for a total of $5x10^4$ cells/ 100 µL of media per collagen-coated transwell with 500µL of media in the basolateral chamber. The cells were then incubated at 37°C, 5% CO_2 overnight. On Day 1, the media in the apical and basolateral chambers were exchanged with 100 µL and 500 µL, respectively, with fresh PneumaCult™ Ex-Plus media. NHBECs were 80-100% confluent the day after seeding. The next PneumaCult™ Ex-Plus media change was on Day 3. On Day 4-7, or until culture reached confluency, the apical and basolateral chambers were fed PneumaCult™ ALI media (StemCell #05021) daily supplemented with 10µM ROCK inhibitor (Tocris Y-27632). On Day 8, the apical media was removed, and the basal media replaced with PneumaCult™ ALI media without Y-27632. Subsequent media changes were every other day thereafter. On Day 14 post-airlift, the apical surfaces were washed with DPBS, once per week. Cells were grown in 37°C, 5% CO_2 incubator until four weeks post airlift.

Directed differentiation of hESC and hiPSCs to lung organoids (LOs)

For human iPSC-LO generation, we used 6 iPSC cell lines **[Supplementary Table 1]** which were cultured in feeder free conditions upon Matrigel (Corning #354230) coated plates in mTeSR Plus medium (StemCellTech #100-0276). Media was changed daily, and stem cells were passaged weekly using enzyme free dissociation reagent ReLeSR™ (Stem Cell Tech #05872). Cultures were maintained in an undifferentiated state, in a 5% CO_2 incubator at 37°C. For lung progenitor spheroid generation, human PSCs were dissociated into single cells with exposure to 20 min of accutase, and approximately 2.0×10^5 cells were seeded on Matrigel-coated plates in mTeSR supplemented with 10µM Y-27632 and incubated for 24 hours. Each iPSC cell line required seeding density optimization with the goal of 50-70% confluency the day after seeding. Definitive Endoderm (DE) induction medium (RPMI1640 + Glutamax, 2% B27 without retinoic acid supplement, 1% HEPES, 50 U/mL penicillin/streptomycin) supplemented with 100 ng/mL human activin A (R&D), 5 µM CHIR99021 (Stemgent), was added on day 1. On days 2 and 3 cells were cultured in DE media with only 100 ng/mL human activin A supplemented. Anterior Foregut Endoderm (AFE) was generated with serum free basal media (SFBM) made up of 3 parts IMDM:1-part F12, 1% B27 without retinoic acid supplement, 0.5% N2 supplements, 50 U/mL penicillin/streptomycin, 0.25% BSA, 50 µg/mL L-ascorbic acid, 0.45 mM monothioglycerol. SFBM was supplemented with 10 µM SB431542 (R&D) and 2 µM

Dorsomorphin (StemGent) on days 4-6. On day 7, AFE cells were dissociated with accutase exposure for 10 min and 3 x 10^5 cells were passaged as aggregates into 150 μL of cold matrigel droplets to make Lung Progenitor Cell (LPC) Spheroids. The matrigel containing cells were polymerized for 30-60 min in a 37°C incubator and then LPC media (SFBM supplemented with 10 ng/mL of human recombinant BMP4, 0.1 μM of retinoic acid (RA), 3 μM of GSK3β inhibitor/Wnt activator CHIR99021 and 10 μM of Rock Inhibitor Y-27632) was added to submerge the matrigel droplets. LPC media was changed every other day for 9-11 days without Y-27632. Analysis of the surface antigen CPM, or the intracellular marker NKX2-1 should be performed at the end of this differentiation period to determine the efficacy of differentiation. If the LPC spheroids express > 50% CPM/NKX2-1, LO differentiation may proceed.

To generate LOs (proximal, distal, and whole lung), LPC spheroids were incubated in 2 U/ml dispase for 30 min at 37°C with manual pipetting every 15 min to break apart the matrigel. Cold PBS was added, transferred to a 15 mL conical tube, and then centrifuged at 400 x g for 5 min. The supernatant was carefully removed then 3 mL of cold PBS was added to the pellet, mixed, and then centrifuged again at 400 x g for 5 min. The supernatant was carefully removed, and the pellet resuspended in 2-3 mL of TrypLE Express (Gibco # 12605010) for 12 min at 37°C to keep the LPC spheroids as aggregates. The reaction was quenched with 2% FBS in DMEM/F12 then centrifuged at 400 x g for 5 min. The supernatant was removed, and the cell pellet resuspended in 1ml of quenching media supplemented with 10 μM Rock inhibitor (Y-27632) and a cell count was performed. Cells were embedded into cold matrigel as aggregates at 5×10^4 cells per 200 μL of Matrigel and transferred into the apical portion of 0.4 μm translucent 12-well inserts (VWR 10769-208).

To generate 3D human proximal LOs, we modified a previously published protocol. After the LPC spheroids were passaged onto transwells, PLO media was added to the basal side of the insert. The medium was made up of SFBM supplemented with 250 ng/mL FGF2, 100 ng/mL rhFGF10, 50 nM dexamethasone (Dex), 100 μM 8-Bromoadenosine 3',5'-cyclic monophosphate sodium salt (Br-cAMP), 100 μM 3-Isobutyl-1-methylxanthine (IBMX) and 10 μM ROCK inhibitor (Y-27632). PLO media was changed every other day for 2-3 weeks.

To generate 3D human distal LOs, we modified a previously published protocol. After the LPC spheroids were passaged onto transwells, DLO media was added to the basal side of the insert. DLO maturation media was made up of distal basal media (95% F12 media, 0.25% BSA, 15 mM HEPES, 0.8 mM calcium chloride (Wako), 0.1% ITS premix (Corning), 1% B27 without RA and 50 U/mL penicillin-streptomycin) supplemented with 3 μM CHIR99021, 10 μM SB431542, 10 ng/ml FGF7, 0.1 μM RA, 50nM Dex, 100 μM Br-cAMP, 100 μM IBMX and 10 μM ROCK inhibitor (Y-27632). DLO media was changed every other day for 2-3 weeks without Y-27632.

To generate 3D human whole LOs, we modified a previously published protocol. LPCs in matrigel droplets were passaged as above. WLO induction media was added to the basal side of the transwell with SFBM supplemented with 10 ng/mL FGF7, 10 ng/mL FGF10, 10 ng/mL EGF, 3 μM CHIR99021, and 10 μM ROCK inhibitor (Y-27632). Media changes

without Y-27632 were done every other day for 6 days. WLO branching medium was added using SFBM supplemented with 10 ng/mL FGF7, 10 ng/mL FGF10, 10 ng/mL EGF, 3 μM CHIR99021, 0.1 μM retinoic acid and 10 ng/mL VEGF/PIGF for 6 days, with media changes every other day. Finally, WLO maturation medium was added using SFBM supplemented with 10 ng/mL FGF7, 10 ng/mL FGF10, 10 ng/mL EGF, 3 μM CHIR99021, 0.1 μM All-trans retinoic acid, 10 ng/mL VEGF/PIGF, 50 nM Dex, 100 μM Br-cAMP, and 100 μM IBMX for 6 days, with media changes every other day.

For infections in intact 3D LOs, the matrigel droplets were washed with PBS, then 1 ml of Cell Recovery Solution (Corning #354253) was added per 200 μL Matrigel drops. Matrigel was mechanically dissociated using wide bore P1000 tips, titurating gently to maintain the integrity of the organoids. The mixture was placed at 4°C for 1 h, with manual resuspension every 15 min. After 1 hour, the sample was transferred to a 15 mL conical tube, using a wide bore pipette. The organoids were centrifuged at 200 x g for 1 min. Chilled PBS was added to the pellet and gently mixed then centrifuged at 200 x g for 1 min. The pellet was resuspended in 500 μL of LO media supplemented with 10 μM Y-27632 in 24 well ultra-low attachment (ULA) plate (Corning #3473) at approximately 20-30 organoids per well. On the day of infection, the 3D organoids were placed in LO medium without DCI.

For infections in 2D monolayers, 3D LOs were dissociated into single cells and seeded at $2x10^4$ cells per well of a matrigel coated 96-well plate approximately 3 days before infection. Matrigel containing LOs was incubated in 2 U/ml dispase for 30 min at 37 °C with manual pipetting every 15 min to break apart the matrigel. Cold PBS was added to the well, transferred to a 15 mL conical tube and then centrifuged at 400 x g for 5 min. The supernatant was carefully removed then 3 mL of cold PBS was added to the pellet, resuspended, and then centrifuged again at 400 x g for 5 min. The supernatant was carefully removed, and the pellet resuspended in 2-3 mL of TrypLE Express (Gibco # 12605010) for 15-20 min at 37°C to dissociate the organoids into single cells. The reaction was quenched with 2% FBS in DMEM/F12 then centrifuged at 400 x g for 5 min. The supernatant was removed, and the cell pellet resuspended in 1ml of quenching media supplemented with 10 μM Rock inhibitor (Y-27632) and a cell count was performed. Cold PBS was added to the mixture then centrifuged at 400 x g for 5 min. Supernatant was carefully removed and resuspended in 2-3 mL of TrypLE Express (Gibco # 12605010) for 20 min at 37 °C. Reaction was quenched with 2% FBS in DMEM/F12 then centrifuged at 400 x g for 5 min. The supernatant was aspirated, and the cell pellet resuspended in 1 mL of quenching media supplemented with 10 μM Rock inhibitor (Y-27632). Cell count was performed, and 2×10^4 cells were seeded per well of a matrigel coated 96-well plate with 100 μL of respective LO media. The wells were 90-100% confluent prior to infections. On the day of infection, the media was replaced with LO medium without DCI.

All materials used can be found in **Supplementary Table 4**. All media recipes can be found in **Supplementary Table 5.**

Infection by Pseudotyped virus

Vesicular Stomatitis Virus (VSV) pseudotyped with spike proteins of SARS-CoV-2 were generated according to a published protocol (35). Briefly, HEK293T, transfected to express the SARS-CoV-2 spike protein, were inoculated with VSV-G pseudotyped ΔG-luciferase or GFP VSV (Kerafast, MA). After 2 h incubation at 37°C, the inoculum was removed and DMEM supplemented with 10% FBS, 50 U/mL penicillin, 50 mg/mL streptomycin, and VSV-G antibody (I1, mouse hybridoma supernatant from CRL-2700; ATCC). Pseudotyped particles were collected 24 h post-inoculation, centrifuged at 1,320 x g and stored at -80°C until use.

For pseudovirus-GFP infections, VSV-G pseudotyped ΔG-GFP was added to 3D or 2D hiPSC derived LOs at an MOI of 1-5 for 24 h. After infection, the cells were washed with 1× Dulbecco's phosphate-buffered saline (PBS) three times. The 3D organoids were prepped for flow cytometry/FACS or single cell RNA sequencing per the protocol below. The monolayer infected lung cells were washed with PBS three times then fixed in 4% PFA for immunohistochemistry (see below).

SARS-CoV-2 viruses

All work with SARS-CoV-2 was conducted in Biosafety Level-3 conditions at the University of California San Diego following the guidelines approved by the Institutional Biosafety Committee.

<u>Isolates:</u>

SARS-CoV-2 isolates WA1 (USA-WA1/2020, A), B.1 (USA/NY-PV08410/2020, B.1, D614G) and Beta (hCoV-19/South Africa/KRISP-K005325/2020, B.1.351, Beta) were acquired from BEI and passaged once through primary human bronchial epithelial cells (NHBECs) differentiated at air-liquid interface (ALI) to select against furin site mutations. The virus was then expanded by one passage through TMPRSS2-Vero cells.

Alpha (hCoV-19/USA/CA_UCSD_5574/2020, B.1.1.7, Alpha) was isolated from a patient nasopharyngeal swab under UC San Diego IRB #160524. The original clinical isolate sequence can be accessed at GISAID accession name: EPI_ISL_751801. Material from nasopharyngeal swab in PBS with 1x Antibiotic-Antimycotic (Thermo Fisher Scientific #15240062) was added to the apical chamber of 4 weeks old ALI NHBECs after two 30 min PBS washes at 37˚C. 1 x Antibiotic-Antimycotic was also added to the media in basal chambers. After 24 h, the material was removed, and apical surfaces were washed. Apical washes (passage 1) were taken daily and stored at -80. Fresh NHBECs at ALI were inoculated with passage 1 virus as above to produce passage 2 and with passage 2 to produce passage 3. Titers were expanded by infecting TMPRSS2-Vero cells with passage 3 virus. Gamma (hCoV-19/Japan/TY7-503/2021, P.1, Gamma), and Delta (hCoV-19/USA/PHC658/2021, B.1.617.2, Delta) were acquired from BEI and expanded on TMPRSS2-VeroE6 cells.

Omicron (hCoV-19/USA/CA-SEARCH-59467/2021, BA.1, Omicron) was isolated from a patient sample under UC San Diego IRB #160524. Original patient isolate sequence can be found at GISAID (EPI_ISL_8186377). Serial dilutions in DMEM + 3% FBS were made from a positive nasopharyngeal swab stored in viral transport media. Dilutions were incubated on TMPRSS2-VeroE6 cells and monitored for CPE. When CPE was observed, the contents were transferred to fresh cells for a total of 3 passages on TMPRSS2-VeroE6 cells.

For single cell RNAseq of the mixed organoids, SARS-CoV-2 isolate WA1 (USA-WA1/2020, A) was acquired from BEI and propagated on VeroE6 cells resulting in mutation of the furin cleavage site (142, 143).

All viral stocks were clarified and stored at -80ºC. Titers were determined by plaque assay or fluorescent focus assay on TMPRSS2-VeroE6 cells. All stocks are verified by full genome sequencing. Original sample sequencing links can be found in **Supplementary Table 6.**

Infection of NHBECs at ALI

After two 30 min incubations with PBS at 37°C, 5% CO_2, virus diluted in PBS was added to the apical chamber in 100 µL. Virus was removed after 24 h and apical washes (150 µL PBS with 10 min incubation at 37°C, 5% CO_2,) were taken daily and stored at -80ºC. Titer was determined by fluorescent focus assay on TMPRSS2-Vero cells. Infected transwells were fixed in 4% PFA for immunofluorescence staining.

Plaque assay

Ten-fold serial dilutions were made in DMEM and added to 12-well plates of confluent VeroE6 cells for 1 h at 37˚C with occasional rocking. Input was removed and monolayers were overlaid with 1mL of a 1:1 mixture of 1.2% agarose and 2 x MEM complete (2 x MEM supplemented with 8% FBS, 2 x L-glutamine, 2 x non-essential amino acids, and 2 x sodium bicarbonate). Plates were incubated 48 h at 37˚C and then fixed with 2 mL 10% formaldehyde in PBS for 24 h at RT. Overlays were removed, and plaques were visualized by staining with 0.025% crystal violet in 2% EtOH. Plaque assays were performed and counted by a blinded experimenter. Plaque assays were counted and recorded by a blinded observer.

Fluorescent focus assay

Ten-fold serial dilutions were made in DMEM and added to 96-well plates of confluent TMPRSS2-VeroE6 cells. After 1 h at 37˚C, input was removed, and wells were overlaid with 100 µL 1% methylcellulose in DMEM + 1% FBS and 1 x Pen/Strep. Plates were fixed after 24 h incubation at 37˚C by addition of 150 µL 8% formaldehyde for at least 30 min at RT. Cells were stained with anti-nucleocapsid primary antibody (GeneTex, gtx135357) and anti-rabbit AlexaFluor secondary

(Thermo Fisher Scientific) and plates were imaged on the Incucyte S3 imager. Foci were counted using the Incucyte onboard software tools.

Virus quantification, FFU immunofluorescence imaging and analysis

3D hPSC-LOs were removed from matrigel intact as described above. They were plated in 24 well ultra-low attachment plates in LO base media, without DCI, at a concentration of 20 organoids per well. hPSC-LOs that were dissociated and plated as a monolayer were also changed to LO base media without DCI, prior to infection. 3D LOs were infected with SARS-CoV-2 at an MOI of 1-3 and incubated for 24 -72 h at 37 °C. Dissociated LOs were infected with SARS-CoV-2 at an MOI of 0.1.

For the compound experiments, 3D LOs in suspension were pretreated with DMSO (control), cathepsin inhibitors ONO5334 and Apilimod and remdesivir (all at a concentration of 5 µM). Two hours after the addition of compounds, SARS-CoV-2 was added for 24 h. Mock infections were performed using viral growth media (DMEM + 3% FBS). Infected and mock controls were washed three times in PBS and lysed in radioimmunoprecipitation assay (RIPA) lysis buffer (Millipore 20-188) at 2 x with protease inhibitor for western blot analysis.

For infections with poractant alfa and recombinant SP-B, hiPSC LOs were dissociated & plated as a monolayer (4 x 10^4 cells/well) (along with VeroE6 cells as a positive control), pre-incubated with exogenous surfactant_(poractant alfa [Curosurf] 1 mg/ml) or with recombinant SP-B [rSP-B] (5 µM) for 30 min prior to infection with SARS-CoV-2 at an MOI of 0.1. After 1 hour of infection, cells were overlayed in media supplemented with 1.2% carboxymethylcellulose as discussed below.

For infections of VeroE6 cells using carboxymethylcellulose, media was prepared using 500 mL LO base media or DMEM-glutamax supplemented with 3% FCS, 2% gentamycin, 1% penicillin/streptomycin plus 6 g autoclaved carboxy methyl cellulose. iPS-derived human LOs were dissociated and plated as a monolayer then infected with SARS-CoV-2 at the indicated MOI. After 1 h of infection, cells were overlayed in carboxymethylcellulose media. After incubation for up to 72 h, overlay was removed and cells were fixed in 4% formaldehyde for 30 min at RT. Fixed cells were washed in BD CytoPerm, and then stained overnight with a primary nucleocapsid antibody (GeneTex GTX135357), counterstained with an anti-rabbit AF647 secondary antibody (ThermoFisher), and nuclei counterstained with 1 µM Hoechst or 1 µM Sytox Green.

Whole well scans were captured with an Incucyte S3 or 10 x 0.45 NA objective using a Nikon Ti2-E microscope with a Qi-2 camera and Nikon Elements 5.02.02 acquisition software utilizing the Jobs module. Wells were imaged for red, green and/or far-red fluorescence using a SpectraX light engine (Lumencor) with individual LFOV filter cubes (Semrock) (excitation/emission maxima at 554/609, 470/525 and 618/698 nm, respectively). FFU analysis was carried out using a custom macro written in the Fiji distribution of ImageJ. The macro leverages machine learning using Ilastik (144), StarDist

(arXiv:1806.03535v2) and GPU acceleration using CLIJ to process data files (145). Nuclei were segmented using StarDist, and positive cell stain masks generated using a trained model in Ilastik. Masks were used to count the number of nuclei in clusters, and to define the staining intensity of the positive clusters, and morphological characteristics and size of FFU, and the distribution of the clusters within the well and from each other. The cluster shape was also assessed by generating a ratio of the perimeter and convex hull of each cluster, with values approaching 1 being circular and values approaching zero becoming more irregular.

FFUTrackR data visualization

Image J datalogs from analyses of whole well scans were visualized using the R Shiny app FFUTrackR (https://croker.shinyapps.io/ffutrackr). The FFUTrackR pipeline was developed using Shiny in R 3.6.3., the R packages Tidyverse and plotrix for basic data loading and filtering utilities. shinydashboardPlus and shinyWidgets are used for module management and custom input controls. FFUTrackR requires R packages dplyr, ggpubr, and ggbeeswarm for basic data handling and plotting utilities. The code for FFUTrackR is available at: https://github.com/WeiqiPeng0/FFU.TrackR.

Virus-induced cell death

SARS-CoV-2-triggered cell death of Vero E6 cells was monitored using an Incucyte S3. Virus was added to Vero E6 cells at the indicated MOI for 1 h in DMEM/10% FBS at 37ºC/5%CO_2, then BH3 mimetics added for up to 48 h. Cell viability was monitored using 1 µg/mL propidium iodide. Five fields of view at 10x magnification representing 33.6% well coverage was monitored for changes in cell viability every 6 h. PI-positive cells were identified using Incucyte software.

Bulk RNA sequencing preparation and analysis:

RNA was purified from TRIzol lysates using Direct-zol RNA Microprep kits (Zymo Research R2061) according to manufacturer recommendations that included DNase treatment. RNAseq assay was performed using an Illumina NEXTSeq 500 platform in the SBP Genomics Core. Briefly, PolyA RNA was isolated using the NEBNext® Poly(A) mRNA Magnetic Isolation Module and barcoded libraries were made using the NEBNext® Ultra II™ Directional RNA Library Prep Kit for Illumina® (NEB, Ipswich MA). Libraries were pooled and single end sequenced (1X75) on the Illumina NextSeq 500 using the High output V2 kit (Illumina Inc., San Diego CA).

For viral RNA analysis, sequencing reads were aligned to the SARS-CoV-2/human/USA/WA-CDC-WA1/2020 genome (GenBank: MN985325.1) using Bowtie2 and were visualized using IGV software. After further filtering and quality control, the R package edgeR29 was used to calculate reads per kilobase of transcript per million mapped reads (RPKM) and log2 [counts per million] matrices as well as to perform differential expression analysis. PCA was performed using log2 [counts per million] values and gene set analysis was run with WebGestalt30. Heatmaps and bar plots were generated

using Graphpad Prism software, v.7.0d. In the volcano plots, differentially expressed genes (P-adjusted value < 0.05) with a log2 [fold change] > 1 are indicated. Read data was processed in BaseSpace (basespace.illumina.com). Reads were aligned to Homo sapiens genome (hg19) using STAR aligner (https://code.google.com/p/rna-star/) with default settings.

We used Cutadapt v2.3 [147] to trim Illumina Truseq adapters, polyA, and polyT sequences. Trimmed reads were first aligned to SARS-CoV-2 genome version NC_045512v2 using STAR aligner v2.7.0d_0221 [148] with parameters according to ENCODE long RNA-seq pipeline (https://github.com/ENCODE-DCC/long-rna-seq-pipeline). Unaligned reads to SARS-CoV-2 genome were subsequently mapped to human genome hg38 using STAR and parameters as above. Gene expression levels were quantified using RSEM v1.3.1 [149] and Gencode gene annotations v32 (Ensembl 98). RNA-seq sequence, alignment, and quantification qualities were assessed using FastQC v0.11.5 (https://www.bioinformatics.babraham.ac.uk/projects/fastqc/) and MultiQC v1.8 [150]. Biological replicate concordance was assessed using principal component analysis (PCA) and pairwise Pearson correlation analysis. Lowly expressed genes were filtered out by applying the following criterion: estimated counts (from RSEM) ≥ number of samples * 5. Filtered estimated read counts from RSEM were compared using the R Bioconductor package DESeq2 v1.22.2 based on generalized linear model and negative binomial distribution [151]. Genes with Benjamini-Hochberg corrected p-value < 0.05 and fold change ≥ 2.0 or ≤ -2.0 were selected as differentially expressed genes. Differentially expressed genes were then analyzed using Ingenuity Pathway Analysis (Qiagen, Redwood City, USA) and Gene Set Enrichment Analysis (GSEA) (146).

Single-cell lung organoid preparation for scRNA-seq

3D hPSC-LOs in ultra-low attachment plates were dissociated into single cells using accutase (Gibco) at 37 °C for 10 min, 24hpi. The dissociated organoids were pelleted and resuspended with DMEM F12 + 10% FBS. The resuspended organoids were then placed through a 40 μm filter to obtain a single-cell suspension. To remove the dead cells, 1 μL Dead Cell Removal cocktail (Annexin V, STEMCELL Technology) was added into the dissociated CO/PCCO single cells suspension together with 1 μL Biotin selection cocktail (Annexin V, STEMCELL Technology), gently mixed and incubated at RT for 3 min. After 3 min incubation, RapidSpheresTM (STEMCELL Technology) was vortexed for 30 s at RT and 2 μL added to the above mixture. At the same time, 850 μL LO base medium was added and gently pipetted 2~3 times. Tubes were then placed into a magnetic holder and incubated for 3 min at RT. After incubation, the supernatant was collected in a new tube for 10X GEM generation. For each of the SC-RNA-seq library, we pooled 20 individual LOs derived from H9 cells and iPSC-lines specified in the manuscript.

For the hPSC LOs outside the BSL3, they were dissociated from matrigel as described above using dispase and trypsin. The resuspended organoids were then placed through a 40 μm filter to obtain a single-cell suspension and stained

with DAPI followed by sorting of live cells using the FACS Aria Fusion Flow Cytometer (BD Biosciences). The sorted cells were washed with 1× PBS + 0.04% BSA, counted manually after sorting and the cell viability was assessed with trypan blue.

Single cell library preparation and sequencing

10X sc-RNA-seq-3'-V3.1 kit (10X Genomics) was used to generate the GEM, cDNA, and sequencing library according to the manufacturer's instructions (10X Genomics). Briefly, live cells were partitioned into nanoliter-scale Gel Bead-In-Emulsions (GEMs) with the 10x Chromium Controller (10X Genomics), 1000 cells were targeted. Upon cell lysis and dissolution of the Single Cell 3′-V3.1 Gel Bead within the droplet, primers containing an Illumina P7 and R2 sequence, a 14 bp 10XBarcode, a 10 bp randomer, and a poly-dT primer sequence were released and mixed with the cell lysates and bead-derived Master Mix. Barcoded, full-length cDNA from poly-adenylated mRNA was then generated in each individual bead, then individual droplets were broken and homogenized before the remaining non-cDNA components were removed with silane magnetic beads (Invitrogen). The libraries were then size-selected, and the R2, P5 and P7 sequences were added to each selected cDNA during end repair and adapter ligation. After Illumina bridge amplification of cDNA, each library was sequenced with the PE100 reads on the Novaseq6000 at the IGM Core in UCSD (mixed LO samples), and The Scripps Oceanography Genomics Core (PLO samples), to a depth of approximately 120M reads per sample.

The FASTQ files were imported to a 10x Cell Ranger data analysis pipeline (v.3.0.2) to align reads, generate feature–barcode matrices and perform clustering and gene expression analysis. In the first step, cellranger mkfastq demultiplexed samples and generated fastq files; and in the second step, cellranger count aligned fastq files to the reference genome and extracted gene expression unique molecular identifier (UMI) count matrix. To measure viral gene expression in LOs, we built a custom reference genome by integrating the four virus genes and GFP into the 10x pre-built human reference (GRCh38 v.3.0.0) using cellranger mkref. The sequences of four pseudo-viral genes (VSV-N, VSV-P, VSV-M and VSV-L) and SARS-CoV-2 genes (E, M, N, S, ORF1ab, ORF3a, ORF6, ORF7a, ORF8, and ORF10) were retrieved from NCBI https://www.ncbi.nlm.nih.gov/nuccore/335873).

scRNA-seq data analysis for lung organoids

We prepared a combined human genome version hg38 and SARS-CoV-2 genome version NC_045512v2 for alignment of 10X scRNA-seq raw data. We used human Gencode gene annotations v32 (Ensembl 98) augmented with SARS-CoV-2 predicted genes from NCBI for the Cell Ranger processing step. scRNA-seq reads from each sample were aligned to the combined genome using Cell Ranger v5.0.1. Data QC, filtering, integration, clustering, and differential expression were performed using Seurat v4.0.5 (147) and Harmony (148). Prior to integration of samples, cells with <40%

mitochondrial content and < 98 percentile of genes detected (to remove potential doublets/multiplets) were kept in each sample for downstream analysis. The samples were merged and gene counts for the merged dataset were normalized using *NormalizeData()*. 3000 variable features were determined using *FindvariableFeatures()* and the data scaled using *ScaleData()*. PCA components were computed using *RunPCA()* and subsequently the samples were integrated using *RunHarmony()* function. Clusters of cells were computed using *RunUMAP(reduction="harmony," dims=1:40)*, *FindNeighbors(reduction="harmony", dims=1:40)*, and *FindClusters(resolution=0.3).* Cluster markers were found using *FindAllMarkers().* Infection and IFNome scores were computed using *AddModuleScore().* Differential expression analyses were performed using *FindMarkers()*. Feature, violin, and other plots were prepared using Seurat or ggplot2. Pathway analyses of differentially expressed genes were performed using Ingenuity Pathway Analysis (Qiagen, Redwood City, USA) and Gene Set Enrichment Analysis (GSEA) (146).

To obtain a list of top interferon-stimulated genes (ISGs), all human type I interferon datasets were taken from the Interferome v2.0 database (87). For each dataset, significantly up-regulated genes (log2 fold change > 1; unadjusted p-value < 0.05) were ranked by the magnitude of their log2 fold changes. These ranks were then combined across datasets by geometric mean using the TopKLists (149) R package to produce an overall ranked list of type I ISGs (Interferon Stimulated Genes) identified.

Identifying & Labeling Endosomes (Macropinosomes)

Endosomes (specifically macropinosomes) were labeled and quantitated utilizing previously established procedures (150-152). Macropinosomes in live cells were labelled by their uptake of high molecular weight fluorescent dextran (FITC-Dextran). In brief, live cells are incubated with 1 mg/mL high molecular weight FITC-Dextran for 30 min, washed with cold PBS, fixed with 3.7% paraformaldehyde and nuclei stained with DAPI. Quantitation was as previously described using image analysis facilitated by Cytation 5 automated imaging and the Gen 5 software. Macropinocytosis in lung cells is also dependent on PI3K; uptake in these cells was inhibited by treatment with LY294002 (25 mM), an inhibitor of PI3K. Cells were pre-treated with the respective inhibitors for 0.5-3 hours prior to infection. At least 25,000 cells-per condition were analyzed for n=3 replicates.

Western blot

Cell samples were washed 3 times with PBS to remove traces of media components, pelleted and lysed with ice cold RIPA buffer containing protease and phosphatase inhibitors (Thermoscientific Halt kit). Samples were vigorously vortexed and centrifuged at 16,000 x g for 25 min at 4°C to separate the proteinous supernatant from the cellular debris. The protein concentration of the supernatant was calculated using a BCA assay kit (ThermoScientific, 23225). 10 μg of

protein and Nupage sample buffer with diothiothreitol was boiled at 95°C for 3 min then loaded into a bis-tris gel and run with MES running buffer. The gel was transferred to 0.45 µm PVDF membrane for 60 min at 20V, then blocked in 5% w/v nonfat dry milk (NFDM) in tris buffered saline (TBS) for 60 min. Primary antibodies were diluted in 1% NFDM in TBS with 0.1% Tween-20 (TBST) and incubated overnight at 4°C. The membranes were then washed in TBST 3 times for 5 min on an orbital shaker. Secondary antibodies (LICOR goat anti rabbit 800CW and goat anti-mouse 680, 1:10,000) were diluted in 1% NFDM for 60 min then rinsed with TBST before imaging on the odyssey near infrared scanner.

Immunofluorescence

Dissociated lung cells were washed twice in PBS, then fixed in BD Fix/Perm containing 4% formaldehyde for 30 min at RT. Fixed cells were washed twice in BD Cytofix/Cytoperm and stained for SARS-CoV-2 with a primary Nucleocapsid antibody at 1:2000 (GeneTex GTX135357) conjugated to AF594 (ThermoFisher A20185) and nuclei counterstained with Hoechst or Sytox Green at 1:5000. 3D Organoids were fixed in 4% paraformaldehyde for 1 h, then washed twice with PBS. Fixed organoids were embedded in a 30% w/v solution of sucrose to preserve the fine cytoarchitecture from cryo-damage. Organoids were then imbedded into OTC and sliced at 10 µm. Serial sections were mounted onto glass slides and allowed to air dry before staining. Sections were incubated in primary antibody diluted in 5% v/v donkey serum and 5% w/v bovine serum albumin with TX-100 for 24 h at 4°C then secondary antibodies at room temperature for 1 h. Nuclei were counterstained by DAPI (1:5000). The information for the primary and secondary antibodies is provided in **Supplementary Table 7**. The figures were processed using ImageJ software.

Intracellular flow cytometry analysis

Flow cytometry intracellular staining was performed following the instruction manual for the Fixation/Permeabilization Solution Kit (BD Biosciences). In brief, infected cells were washed twice with PBS, then incubated in Zombie UV (BioLegend 423107) per the manufacturer’s recommendations for 30 min at room temperature. Cells were washed in FACS buffer (PBS + 2% FBS) once and resuspended in Fixation/Permeabilization solution at 4 °C for 30 min. The cells were washed twice in 1× Perm/Wash buffer, incubated with primary Nucleocapsid antibody (GeneTex GTX135357) conjugated to AF594 (ThermoFisher A20185) at 4 °C for 30 min in the dark, and washed twice before flow cytometry analysis. Flow cytometry was performed using mock infected LOs as a negative control and gates were set to exclude the dead cells (Zombie UV stained) using the LSR Fortessa X-20 (BD Biosciences). All experiments were done a minimum of three separate times in three technical replicates. Data analysis was performed using FlowJo software and statistical analyses were done in Prism 8 (GraphPad). The information for the primary antibodies and antibodies is provided in **Supplementary Table 7.**

Poractant Alfa and recombinant SP-B

Vero cells were plated in a monolayer in a 96 well plate and 3 titrations (1 mg/mL, 0.1 mg/mL, and 0.01 mg/mL) of Poractant alfa (Curosurf) were added 60 min prior to infection. The SARS-CoV-2 variants (Alpha, Beta, Gamma, Delta, WA, and NY) were added at 150 FFU and the plate was placed on a shaker for 1 h. The plate was then overlayed with 150 µL of DMEM/Carboxymethylcellulose/FBS mix and placed in the incubator for 24 h. The hPSC derived LOs were plated as a monolayer in a 96 well plate as previously described. They were exposed to 1 mg/mL of Poractant Alfa or 6.6 µM of recombinant SP-B (Prospec #PRO-2585) for 60 min prior to infection. SARS-CoV-2 was added at an MOI of 0.1 and the plate was placed on a shaker for 1 h. The plate was then overlayed with 150 µL of SFBM/Carboxymethylcellulose/FBS mix and placed in the incubator for 24 h. After 24h, the plates were washed twice with PBS and fixed and stained as per the immunofluorescence protocol above.

Quantification And Statistical Analysis

Statistical analysis was performed with Prism 8 (GraphPad Software). We compared all time course data related to cell death by two-way analysis of variance (ANOVA) followed by a Šidák multiple comparisons test. For the poractant alfa and rSP-B experiments, a one-way ANOVA or one-tailed t-test was used to determine significance. This was followed by a Šidák multiple comparisons test correction.

III. DETAILED ACKNOWLEDGMENTS

We thank the UC San Diego Center for Advanced Laboratory Medicine Clinical Microbiology and Virology Lab and UC San Diego EXCITE for providing clinical samples for viral isolation and SARS-CoV-2 genome sequencing.

The following reagent was deposited by the Centers for Disease Control and Prevention and obtained through BEI Resources, NIAID, NIH: SARS-Related Coronavirus 2, Isolate USA-WA1/2020, NR-52281. The following reagent was obtained through BEI Resources, NIAID, NIH: SARS-Related Coronavirus 2, Isolate New York-PV08410/2020, NR-53514. The following reagent was obtained through BEI Resources, NIAID, NIH: SARS-Related Coronavirus 2, Isolate hCoV-19/South Africa/KRISP-K005325/2020, NR-54009, contributed by Alex Sigal and Tulio de Oliveira. The following reagent was obtained through BEI Resources, NIAID, NIH: SARS-Related Coronavirus 2, Isolate hCoV-19/Japan/TY7-503/2021 (Brazil P.1), NR-54982, contributed by National Institute of Infectious Diseases. The following reagent was obtained through BEI Resources, NIAID, NIH: SARS-Related Coronavirus 2, Isolate hCoV-19/USA/PHC658/2021 (Lineage B.1.617.2; Delta Variant), NR-55611, contributed by Dr. Richard Webby and Dr. Anami Patel. The CRISPR corrected hiPSC lines from the SP-B mutant patient generously provided by Dr. Darrell Kotton. Lines GM23450 & GM23720 were provided from the NIGMS Human Genetic Cell Repository at Coriell.

IV. REFERENCES

82. J. R. Wright, Immunomodulatory functions of surfactant. *Physiol Rev* **77**, 931-962 (1997).
83. M. Numata, H. W. Chu, A. Dakhama, D. R. Voelker, Pulmonary surfactant phosphatidylglycerol inhibits respiratory syncytial virus-induced inflammation and infection. *Proc Natl Acad Sci U S A* **107**, 320-325 (2010).
84. R. G. Spragg *et al.*, Effect of recombinant surfactant protein C-based surfactant on the acute respiratory distress syndrome. *N Engl J Med* **351**, 884-892 (2004).
85. D. R. Voelker, M. Numata, Phospholipid regulation of innate immunity and respiratory viral infection. *J Biol Chem* **294**, 4282-4289 (2019).
86. U. Mirastschijski, R. Dembinski, K. Maedler, Lung Surfactant for Pulmonary Barrier Restoration in Patients With COVID-19 Pneumonia. *Front Med (Lausanne)* **7**, 254 (2020).
87. S. Wang *et al.*, The Role of Pulmonary Surfactants in the Treatment of Acute Respiratory Distress Syndrome in COVID-19. *Front Pharmacol* **12**, 698905 (2021).
88. K. Pramod *et al.*, Surfactant-based prophylaxis and therapy against COVID-19: A possibility. *Med Hypotheses* **143**, 110081-110081 (2020).
89. Z. G. Dessie, T. Zewotir, Mortality-related risk factors of COVID-19: a systematic review and meta-analysis of 42 studies and 423,117 patients. *BMC Infect Dis* **21**, 855 (2021).
90. M. Webb Hooper, A. M. Nápoles, E. J. Pérez-Stable, COVID-19 and Racial/Ethnic Disparities. *Jama* **323**, 2466-2467 (2020).
91. A. Wang *et al.*, Single-cell multiomic profiling of human lungs reveals cell-type-specific and age-dynamic control of SARS-CoV2 host genes. *Elife* **9** (2020).
